# Supplementary material for: Fast and quantitative 2D and 3D orientation mapping using Raman microscopy
Source: Nat Commun. 2019 Dec 5;10:5555. doi: 10.1038/s41467-019-13504-8 (PMC6895231; doi:10.1038/s41467-019-13504-8)
Supplement: Supplementary file 1 — Supplementary Information [file 41467_2019_13504_MOESM1_ESM.pdf]

Supplementary Information

Fast and quantitative 2D and 3D orientation mapping using Raman microscopy

O. Ilchenko et al.

Supplementary Note 1: Raman tensor analysis

Raman scattering in crystalline media is described by second-rank symmetric tensor $\Re$ [1]. Intensity of Raman scattered light is dependent on polarization of incident and scattered photons, and also on orientation of a crystal in space. It means that sample crystallographic orientations can be determined by the investigation of Raman scattering anisotropy. In turn, anisotropic parameters can be determined by the measuring of Raman intensity passed through the analyzer in the Raman scattering beam.

Raman scattering intensity I of the phonon mode in the case of back scattering Raman setup is given by the following equation[1]:

$$I \sim \left| e_i^T \, \Re \, e'_s \right|^2, \tag{1}$$

$$\mathbf{e}_i = \begin{pmatrix} \cos\psi_i \\ \sin\psi_i \\ 0 \end{pmatrix}, \qquad \mathbf{e}'_s = \mathbf{M}\,\mathbf{e}_s = \mathbf{M}\begin{pmatrix} \cos\psi_s \\ \sin\psi_s \\ 0 \end{pmatrix}. \tag{2}$$

where $\mathbf{e}_i$ and $\mathbf{e}_s$ are the unit vectors of the polarization of the electric field for incident and scattered irradiation, respectively. In order to describe Raman intensities in off-axis registration geometry, vector $\mathbf{e}_s$ is multiplied by rotation matrix $\mathbf{M}$ which result in $\mathbf{e}'_s$ (in the case of on-axis registration $\mathbf{e}'_s = \mathbf{e}_s$.) Matrix $\mathbf{M}$ depends on off-axis tilt angle β defining rotation of scattered light collection optics around laboratory x axis

$$\mathbf{M} = \begin{pmatrix} 1 & 0 & 0 \\ 0 & \cos\beta & -\sin\beta \\ 0 & \sin\beta & \cos\beta \end{pmatrix}. \tag{2a}$$

Elements of Raman scattering tensor are usually defined in crystal coordinate system, and should be transformed with the change of reference coordinate system. In order to move from crystal related coordinate system (X,Y,Z) to the laboratory coordinate system (x,y,z) we need to apply a rotation matrix $\mathbf{\Phi}(\theta,\phi,\psi)$:

$$\Re_j(x,y,z) = \mathbf{\Phi}(\theta,\phi,\psi)\Re_j(X,Y,Z)\mathbf{\Phi}^{-1}(\theta,\phi,\psi). \tag{3}$$

Here $\mathbf{\Phi}^{-1}(\theta,\phi,\psi)$ is an inverse matrix; (θ,ϕ,ψ) are the Euler angles, j is an index of phonon mode under consideration. Euler angles vary in the following ranges: $0 \le \theta \le \pi$, $0 \le \phi \le 2\pi$, $0 \le \psi \le \pi$.

For Euler angles defined as in reference[2] rotation matrix has the form:

$$\mathbf{\Phi}(\theta,\phi,\psi) = \begin{pmatrix} \cos\theta\cos\phi\cos\psi - \sin\phi\sin\psi & -\sin\phi\cos\psi - \cos\theta\cos\phi\sin\psi & \sin\theta\cos\phi \\ \cos\theta\sin\phi\cos\psi + \cos\phi\sin\psi & \cos\phi\cos\psi - \cos\phi\sin\phi\sin\psi & \sin\theta\sin\phi \\ -\sin\theta\cos\psi & \sin\theta\sin\psi & \cos\theta \end{pmatrix} \tag{4}$$

Owing to orthogonality of rotation matrix $\mathbf{\Phi}$ its inverse can be found by simple transposition:

$$\boldsymbol{\Phi}^T(\theta,\phi,\psi)=\begin{pmatrix} \cos\theta\cos\phi\cos\psi-\sin\phi\sin\psi & \cos\theta\sin\phi\cos\psi+\cos\phi\sin\psi & -\sin\theta\cos\psi \\ -\sin\theta\cos\psi-\cos\theta\cos\phi\sin\psi & \cos\phi\cos\psi-\cos\theta\sin\phi\sin\psi & \sin\theta\sin\psi \\ \sin\theta\cos\phi & \sin\theta\sin\phi & \cos\theta \end{pmatrix}$$

(5)

Finally, Raman intensity in Supplementary Equation 1 can be written as:

$$I(\theta,\phi,\psi;\psi_i,\psi_s)\sim\sum_j\left|\boldsymbol{e'}_s^T\boldsymbol{\Phi}^T(\theta,\phi,\psi)\Re_j(X,Y,Z)\boldsymbol{\Phi}(\theta,\phi,\psi)\boldsymbol{e}_i\right|^2, \quad (6)$$

where summation occurs over the all degenerate phonon modes j and order of matrix multiplication is reversed.

Supplementary Note 2: Si Raman tensor model development

In order to develop a Raman orientation mapping model for Si analysis we need to apply Supplementary Equation 3 to the Si case. Silicon belongs to O_h crystal class, and its sole Raman active phonon mode is F_{2g}. Since it is triply degenerated, Raman intensity is determined by summation over three modes represented by the Raman tensors[7]

$$\Re_x=\begin{pmatrix}0&0&0\\0&0&d\\0&d&0\end{pmatrix},\quad \Re_y=\begin{pmatrix}0&0&d\\0&0&0\\d&0&0\end{pmatrix},\quad \Re_z=\begin{pmatrix}0&d&0\\d&0&0\\0&0&0\end{pmatrix}. \quad (19)$$

Here d is a material constant. Tensor $\Re_x$ corresponding for the longitude crystal mode LO, tensors $\Re_y$, $\Re_z$ corresponding for the transversal crystal modes, TO1 and TO2, respectively.

$$\begin{aligned} &I_{TO1}(\theta,\phi,\psi;\psi_i,\psi_s)=\sin^2\theta(2\cos\theta\sin\phi\cos(\psi+\psi_i)\cos(\psi+\psi_s)+\cos\phi\sin(2\psi+\psi_i+\psi_s))^2 \\ &I_{TO2}(\theta,\phi,\psi;\psi_i,\psi_s)=\sin^2\theta(\sin\phi\sin(2\psi+\psi_i+\psi_s)-2\cos\theta\cos\phi\cos(\psi+\psi_i)\cos(\psi+\psi_s))^2 \\ &I_{LO}(\theta,\phi,\psi;\psi_i,\psi_s)=(\cos^2\theta\sin(2\phi)\cos(\psi+\psi_i)\cos(\psi+\psi_s) \\ &+\cos\theta\sin(2\phi)\sin(2\psi+\psi_i+\psi_s)-\sin(2\phi)\sin(\psi+\psi_i)\sin(\psi+\psi_s))^2 \end{aligned} \quad (20)$$

Supplementary Note 3: Orientation determination error

In order to get a quantitative answer with regard to the orientation determination error, we simulated a testing dataset of Raman intensity variation versus ψ. The dataset consists of intensity values calculated for three classes corresponding to silicon wafer cuts (100), (110) and (111). Each orientation class has 1440 positions of wafer rotation angle ψ in the interval $0°$-$360°$ taken with step 0.25°.

The orientation determination results were obtained by fitting of Euler angles to simulated data and were represented in a crystallographic color code using MTEX toolbox for Matlab[8,9], see Supplementary Figure 5. The color corresponds to orientation of the wafer normal direction (ND) relative to crystallographic axes of silicon. Misorientation angle, which corresponds to

undetermined rotation of the sample, is depicted in Supplementary Figure 5 as vertical bars on the right side of each subplot.

Orientation fitting was done using varying number of polarized channels from 2 to 12 channels, see subplots in Supplementary Figure 5. Analysis of simulation results reveals, that basic orientation determination become possible for four or more channels. However, for some specific orientations, intensity data remains ambiguous and rotation angle ψ is not recovered. This is clearly seen for (111) wafer in misorientation plots in Supplementary Figure 5, where misorientation angle varies from zero to maximal possible for cubic symmetry angle 62.8°. Nevertheless, even for such ambiguous data, some orientation data is still recovered, and it is possible to clearly distinguish wafers (100), (110), (111) even for four measurement channels.

According to simulation, the ambiguity can be successfully resolved when off-axis channels are added to the measurement setup. Still, adding only two off-axis channel is not enough to resolve ambiguity completely. Full resolution become possible when adding three off-axis channels to six on-axis channels, resulting in total nine measurement channels. Combination of polarizations used in this simulation for different number of channels is in correspondence to Fig. 1c. Further addition to number of channels up to 12 does not noticeably increase accuracy of fit, so we decided to use nine channels in our experimental setup as reasonable minimum ensuring unambiguous measurements.

Supplementary Note 4: Analysis of ambiguities in Raman orientation mapping

Simulation performed in Section 4 revealed that some orientations, like those appearing in surface plane (111) of silicon, prone to introduce ambiguity in measurement data. Although simulation helped to define minimal number of polarization channels required to determine orientation, it still has limited scope, because only (100), (110) and (111) cases were considered. To confirm possibility of orientation determination in general case additional analysis was performed.

Ambiguity analysis was done by exhaustive search of indistinguishable solutions over range of all possible Euler angles. Each Euler angle was incremented in 0.5° step and calculated Raman intensities for corresponding orientation were stored in a list. At next step, for each orientation defined by three Euler angles, other orientations having nearest Raman intensities were searched in the list using Euclidian norm as a distance function. Only matches with resulting distance less than predefined threshold 5% of maximal level were selected. Number of matching orientations was recorded as metric of ambiguity. For absolutely unambiguous case, recorded metric should equal to one for all tested orientations. However, such ideal case is never realized it practice, because presence of symmetry elements in tested crystal class make some orientations inherently indistinguishable. Thus in present analysis we are mainly interested in minimal realizable ambiguity metric.

Ambiguity analysis of many polarization combinations containing only on-axis channels quickly revealed, that all of them have ambiguity metric not less than 2. That directly indicates, that each tested orientation have at least one competitive orientation with indistinguishable Raman intensities, but different Euler angles. Quick check showed that this competitive orientation have Euler angle ψ which differs to 180° from true orientation.

Applying off-axis measurement scheme, that means rotating scattered light collecting channels away from direction of irradiation channels, removed $\psi + 180°$ ambiguity. Most of the orientation space in this case have ambiguity metric equal to one, except zones associated with symmetry elements, and some new zones, which appeared due to specific selection of off-axis angle and polarization directions. Using combined scheme, where both on-axis and off-axis channels are present, allowed to minimize volume of orientation space where ambiguity persist.
To verify ambiguity analysis further, test fit was performed. Synthetic 2D map containing grains with different crystal orientations was prepared, and corresponding Raman intensities were calculated. Using simulated intensity data, orientation fit was carried out with different combination of polarization channels (see Table 1). For each channel combination geometric area were fit was erroneous was determined, and presented as percentage of total map area (column "Orientation error" of Table 1). Maximal misorientation angle registered in successfully fitted zones is recorded in column "Misorientation" of the table. The same quantities versus total number of channels are plotted in Supplementary Figure 6.
For number of channels less or equal than 5 orientation of larger part of the map fitted incorrectly. For number of channels from 6 to 8 part of successfully fitted data point approach 50%, that means fit is successful in half of the cases. It is explained by $\psi + 180°$ ambiguity and is the result of mere luck – fitting algorithm correctly guessed orientation only in half of points. Misorientation is still 62.8° (maximal possible in cubic symmetry), because we can not be sure which orientation ψ or $\psi + 180°$ is actually correct. For number of channels greater or equal to 9, which must include off-axis channel, part of incorrectly fitted points is around 1% or less, with minimal misorientation.

Supplementary Note 5: Intensity corrections in SAROM channels

Experimental realization of off-axis scheme (Supplementary Figure 11a) revealed, that obtained measurements are highly sensitive to variations of the sample surface profile when large off-axis angles are used. Defocusing caused by varying surface height results in change of intensity of scattered light. These changes of channels intensities should be compensated before orientation fitting. One possible solution is to use level of fluorescence background for Raman intensity normalization, as being insensitive to crystal orientation.
Another solution, which proved to be also effective, is to rely on relative intensity between channels but not on absolute intensity. To make this possible, fitting procedure should be modified to use normalized values instead of absolute ones. We implemented such normalization as division of intensity in each off-axis channel by Euclidian norm of vector composed of all off-axis intensities. It is done in assumption that all off-axis polarization channels experience the same or similar variation of intensity due to changes in the sample surface profile. The same normalization can also be applied to on-axis channels if they experience uncompensated intensity variation. Our experiments show, that such procedure generally is not required for on-axis channels, but for off-axis channels it allows to efficiently compensate even large intensity variations and obtain reliable orientation fit. Normalization applied to channels which are already well calibrated results in slightly decreased accuracy of the fit, because it actually reduce the effective number of independent channels in group of channels. For the best results, number of channels which are normalized in one group should be at least three or more. Proposed normalization can be applied on different groups of channels independently based on actual quality of registered data.

Supplementary Note 6: Artifacts correction

Geometries and optical coatings in SAROM setup were optimized for the minimum effect of LA and LP anisotropy using Zemax Optics Studio (Supplementary Figure 14). In order to determine the Jones matrix coefficients, we performed numerical polarization analysis and obtained values of phase shift and attenuation between S and P components of the EM field in LBDS and RBDS.

LA and LP anisotropy compensation. During signal propagation through the reflective and transmitting optical elements LA and LP anisotropy usually take place[3]. These effects can be described by the usage of Jones matrix formalism[4], where each optical element is represented by matrix $\mathbf{J}$ with dimensions 3×3.

When the signal is propagating through the multiple optical elements of LBDS described by matrices $\mathbf{J}_1, \mathbf{J}_2, ..., \mathbf{J}_N$, effective matrix can be expressed as:

$$\mathbf{J}_i = \mathbf{J}_N \cdots \mathbf{J}_2 \mathbf{J}_1. \tag{7}$$

During the propagation through the RBDS the matrix will be expressed as:

$$\mathbf{J}_i = \mathbf{J}'_M \cdots \mathbf{J}'_2 \mathbf{J}'_1, \tag{8}$$

where $\mathbf{J}'_1, \mathbf{J}'_2, ..., \mathbf{J}'_M$ describe elements in the path of scattered light with required coordinate transformations applied to represent on-axis or off-axis channel.

Therefore, Raman intensity in Supplementary Equation 1 can be re-written as:

$$I \sim \sum_j \left| \boldsymbol{e'}_s^T \boldsymbol{J}_s^T \boldsymbol{\Phi}^T(\theta, \phi, \psi) \Re_j(X, Y, Z) \boldsymbol{\Phi}(\theta, \phi, \psi) \boldsymbol{J}_i \boldsymbol{e}_i \right|^2, \tag{9}$$

In the case of LBDS, orthogonal laser beams propagate with angle of polarization state: $\psi_i^1 = 0°$, $\psi_i^2 = 45°$, $\psi_i^3 = 90°$. LP anisotropy do not appear at $\psi_i^1 = 0°$ and $\psi_i^3 = 90°$ [5] and present laser intensity attenuation can be experimentally measured and corrected. However, $\mathbf{J}_i$ will acquire more complex view at $\psi_i^2 = 45°$ where LP and LA anisotropy must be taken into account.

Objective NA compensation. Before, we assumed that the laser beam has normal incidence to the sample surface (Supplementary Figure 12). However, we use apertures for on-axis and off-axis Raman scattering collection that have certain NA value. This effect becomes especially critical for high NA objectives[6] relevant for confocal 3D-SAROM measurements. Taking into consideration this angular distribution Raman intensity can be described in the following way:

$$I_\Omega \sim \sum_j \int_{\Omega_i} \int_{\Omega_s} \left| \boldsymbol{e'}_s^T \boldsymbol{J}_s^T \boldsymbol{\Phi}^T(\theta, \phi, \psi) \Re_j(X, Y, Z) \boldsymbol{\Phi}(\theta, \phi, \psi) \boldsymbol{J}_i \boldsymbol{e}_i \right|^2 d\Omega_i d\,\Omega_s, \tag{10}$$

where Ω_i,Ω_s – solid angles of incident and scattering irradiation, respectively. Integration is done within the range of cones formed by optical setup for incident and scattered path.

Raman microscopy require high-power illumination to obtain suitable signal-to-noise ratio, thus the sample is irradiated with coherent laser source. Most of Raman response comes from focus spot, where laser beam have parallel or near-to-parallel wave fronts, which means that actual polarization of irradiating wave does not change much over the registration volume. Thus we can safely drop integration over Ω_i in Supplementary Equation 10 assuming we have irradiation conditions similar to plane-wave case.

Effect of registration of scattered light coming from different angles due to integration over a collecting aperture can be estimated analytically. For uniform intensity response of the collecting lenses, registered intensity is proportional to an integral

$$I_{\Omega_S} \sim \sum_j \int_0^{2\pi} \int_0^{\theta_S} \left| \boldsymbol{e'}_s^T(\theta,\varphi) \Re'_j \boldsymbol{e'}_i \right|^2 \sin\theta \, d\theta \, d\varphi, \tag{11}$$

where θ_S is half-angle of collection aperture. It is convenient to calculate this integral in local spherical coordinate system (Supplementary Figure 15), where *z* axis is oriented along optical axis of collection lens and polarization $\mathbf{e}'_S$ lays in the XOZ plane.

Scattered polarization $\mathbf{e}'_S$ is perpendicular to wave vector $\mathbf{k}_S$ and explicitly depends on integration angles θ and φ. Components of vector $\mathbf{e}'_S$ can be found via rotation matrices

$$\mathbf{e}'_s = \begin{pmatrix} \cos\varphi & -\sin\varphi & 0 \\ \sin\varphi & \cos\varphi & 0 \\ 0 & 0 & 1 \end{pmatrix} \begin{pmatrix} \cos\theta & 0 & \sin\theta \\ 0 & 1 & 0 \\ -\sin\theta & 0 & \cos\theta \end{pmatrix} \begin{pmatrix} \cos\varphi & \sin\varphi & 0 \\ -\sin\varphi & \cos\varphi & 0 \\ 0 & 0 & 1 \end{pmatrix} \begin{pmatrix} 1 \\ 0 \\ 0 \end{pmatrix}. \tag{12}$$

Raman tensor $\Re'$ rotated to local coordinate system and incident polarization vector $\mathbf{e}'_i$ remain constant during integration. Result of their tensor multiplication we define as a vector

$$\mathbf{p}_j = \begin{pmatrix} p_x \\ p_y \\ p_z \end{pmatrix} = \Re'_j \mathbf{e}'_i. \tag{13}$$

To make comparison easier we normalize aperture-integrated intensity on solid angle

$$\Omega_S = \int_0^{2\pi} \int_0^{\theta_S} \sin\theta \, d\theta \, d\varphi = 2\pi(1-\cos\theta_S) \tag{14}$$

Normalized intensity integral (S11) transforms to

$$I_S \sim \frac{1}{2\pi(1-\cos\theta_S)} \sum_j \int_0^{2\pi} \int_0^{\theta_S} \left| \boldsymbol{e'}_s^T \boldsymbol{p}_j \right|^2 \sin\theta \, d\theta \, d\varphi \tag{15}$$

This integral can be computed analytically. For specific angle θ_S total intensity is

$$I_S \sim \sum_j \left(\frac{1}{48}(33+12\cos\theta_S+3\cos 2\theta_S)p_x^2 + \frac{1}{48}(3-4\cos\theta_S+\cos 2\theta_S)p_y^2 + \frac{1}{48}(12-8\cos\theta_S-4\cos 2\theta_S)p_z^2 \right) \tag{16}$$

For small θ_S most intensity comes from term p_x^2, which is in accordance to used selection of local coordinate system. For increasing θ_S coefficient near p_x^2 decreases, and contribution from p_z^2 correspondingly increases. Effect of p_y^2 is much smaller and can be neglected even for as large θ_S angles as 30°.

For transparent samples collection angle value should be corrected for refractive index of a material when signal collection is done from internal sample volume. All samples in this work were collected at aperture with half-angle of 24°. Silicon has quite large index of refraction *n*=3.8 at 785 nm, so internal collection angle decrease significantly comparing to external collection angle and is limited to $\theta_S = 6°$. Corresponding contributions from different components of vector $\mathbf{p}_j$ are

$$I_S \sim \sum_j (0.9971p_x^2 + 1.37\times 10^{-6}p_y^2 + 0.0029p_z^2) \tag{17}$$

Resulting intensity change due to component intermixing will be smaller than 0.3%, so this effect can be safely ignored in Silicon samples. For CBZD and poly-sapphire samples intensity change caused by component intermixing will be around 1.5% due to lower refractive index. For larger collection apertures or samples with lower refractive index collection aperture effect can be fully taken into account by introduction of three multiplicative correction factors applied to the squares of $\mathbf{p}_j$ vector components according to Supplementary Figure 15.

Sample birefringence. Sample birefringence can also leads to the artifacts in the Raman spectra[6]. Depth dependent birefringence correction becomes especially important for 3D-SAROM measurements. Therefore Supplementary Equation 6 will take form:

$$I_\Omega \sim \sum_j \int_{\Omega_i}\int_{\Omega_s} \left| \boldsymbol{e}'^T_s \boldsymbol{J}^T_s \boldsymbol{\Phi}^T(\theta,\phi,\psi) \boldsymbol{X}_s \Re_j(X,Y,Z) \boldsymbol{X}_i \boldsymbol{\Phi}(\theta,\phi,\psi) \boldsymbol{J}_i \boldsymbol{e}_i \right|^2 d\Omega_i d\,\Omega_s, \tag{18}$$

where $\mathbf{X}_{i,s}$ are the birefringence correction matrixes.

Background correction and denoising. Before applying artifact correction procedures described above all Raman spectra in nine polarized channels were background corrected and Savitzky–Golay noise reduction algorithm was applied.

Supplementary Note 7: Poly-Si mapping

The surface of poly- Si is shown on the visible image of Supplementary Figure 16a. We performed mechanical polishing of the sample, in order to reach a surface flatness comparable with that of single-crystal Si wafers. This was a methodologically important step, which helped us to obtain a Raman intensity and reflectivity comparable to those of the single crystal Si. We used a fluorescence intensity map of the surface of the polycrystalline Si plotted at the maximum of Si fluorescence band for Raman intensity calibration (Supplementary Figure 16b). To compensate intensity variations during scanning the sample, we applied intensity normalization procedure described in Section S6 for off-axis group of channels. On-axis channels were initially calibrated using single-crystal samples and weren't re-normalized during processing of Poly-Si data. The polarized Raman maps collected simultaneously from the nine channels are presented in Supplementary Figure 17. The polycrystalline structure of the Si sample can't be seen on the visible microscopy image (Supplementary Figure 16a) due to the polishing, however it can be clearly recognized in the polarized Raman maps (Supplementary Figure 17). Giving a closer look at the data, it is possible to reveal that each single crystal in the polycrystalline sample has different Raman intensity for each polarized channel. The crystals which are not discernible in one polarized channel can be clearly separated in another one.

Due to the high throughput of the SAROM system from the sample to the detector (9.8% of energy for each polarized channel, 88% in all nine channels) and a spectral binning up to 16cm^{-1}, we obtained a high signal-to-noise ratio (SNR) of 1500:1 for each channel in the acquired maps, which is comparable to the SNR ratio of the single-crystal Si (Supplementary Figure 2-4).

Supplementary Note 8: CBZD Raman tensor model development

Monocrystalline CBZD has point group symmetry C_{2h} [10,11]. Therefore, it has two Raman active modes A_g and B_g [1], with the following Raman tensors:

$$\Re_{A_{1g}} = \begin{pmatrix} a & 0 & d \\ 0 & b & 0 \\ d & 0 & c \end{pmatrix}, \quad \Re_{B_g} = \begin{pmatrix} 0 & e & 0 \\ e & 0 & f \\ 0 & f & 0 \end{pmatrix}. \tag{21}$$

a, b, c, d, e, f – material parameters.

According to Supplementary Equation 3 intensity of Raman modes can be expressed as:

$$I_{A_g}(\theta,\phi,\psi,a,b,c,d,\psi_i,\psi_s)=\frac{1}{4}(\cos^2(\theta)\cos(\psi+\psi i)\cos(\psi+\psi s)((a-b)\cos(2\phi)+a+b)-$$
$$2d\sin(2\theta)\cos(\phi)\cos(\psi+\psi_i)\cos(\psi+\psi_s)+2b\sin^2(\psi)\cos(\psi_i)\cos(\psi_s)\cos^2(\phi)+$$
$$2a\sin^2(\psi)\cos(\psi_i)\cos(\psi_s)\sin^2(\phi)+2d\sin(\theta)\sin(2\psi)\cos(\psi_i)\cos(\psi_s)\sin(\phi)-$$
$$2d\sin(\theta)\sin^2(\psi)\sin(\psi_i)\cos(\psi_s)\sin(\phi)+a\sin(2\psi)\sin(\psi_i)\cos(\psi_s)\sin^2(\phi)-$$
$$2d\sin(\theta)\sin^2(\psi)\cos(\psi_i)\sin(\psi_s)\sin(\phi)+a\sin(2\psi)\cos(\psi_i)\sin(\psi_s)\sin^2(\phi)+$$
$$2c\sin^2(\theta)\sin^2(\psi)\sin(\psi_i)\sin(\psi_s)-2\sin(\theta)\sin(\psi)\cos(\psi)(\sin(\psi_s)(c\sin(\theta)\cos(\psi_i)+$$
$$2d\sin(\psi_i)\sin(\phi))+c\sin(\theta)\sin(\psi_i)\cos(\psi_s))+2\cos^2(\psi)(\sin(\psi_i)(a\sin(\psi_s)\sin^2(\phi)+$$
$$b\sin(\psi_s)\cos^2(\phi)+d\sin(\theta)\cos(\psi_s)\sin(\phi))+\sin(\theta)\cos(\psi_i)(c\sin(\theta)\cos(\psi_s)+$$
$$d\sin(\psi_s)\sin(\phi)))-(a-b)\cos(\theta)\sin(2\phi)\sin(2\psi+\psi_i+\psi_s)+b\sin(2\psi)\cos^2(\phi)\sin(\psi_i+\psi_s))^2$$

(22)

$$I_{B_g}(\theta,\phi,\psi,e,f,\psi_i,\psi_s)=\frac{1}{16}(-2\cos(\phi)(4e\sin(\phi)\sin(\psi+\psi_i)\sin(\psi+\psi_s)+f\cos(\theta-2\psi-\psi_i-\psi_s)-$$
$$f\cos(\theta+2\psi+\psi_i+\psi_s))+\cos(\theta)(4e\cos(\theta)\sin(2\phi)\cos(\psi+\psi_i)\cos(\psi+\psi_s)-$$
$$2e\sin(-2\psi-\psi_i-\psi_s+2\phi)+2e\sin(2\psi+\psi_i+\psi_s+2\phi)-f\cos(\theta-2\psi-\psi_i-\psi_s-\phi)+$$
$$f\cos(\theta-2\psi-\psi_i-\psi_s+\phi)-f\cos(\theta+2\psi+\psi_i+\psi_s-\phi)+f\cos(\theta+2\psi+\psi_i+\psi_s+\phi)-$$
$$f\cos(\theta+\psi_i-\psi_s-\phi)+f\cos(\theta+\psi_i-\psi_s+\phi)-f\cos(\theta-\psi_i+\psi_s-\phi)+f\cos(\theta-\psi_i+\psi_s+\phi)))^2$$

(23)

For surface plane (001) Raman intensity has the following view:

$$I_{A_g}(0,0,\psi,a,b,c,d,\psi_i,\psi_s)=\frac{1}{4}\left((a-b)\cos(2\psi+\psi_i+\psi_s)+(a+b)\cos(\psi_i-\psi_s)\right)^2 \quad (24)$$

$$I_{B_g}(0,0,\psi,a,b,c,d,\psi_i,\psi_s)=e^2\sin(2\psi+\psi_i+\psi_s)^2 \quad (25)$$

For surface plane (010) Raman intensity has the following view:

$$I_{A_g}(\frac{\pi}{2},0,\psi,a,b,c,d,\psi_i,\psi_s)=\frac{1}{4}\left((c-b)\cos(2\psi+\psi_i+\psi_s)+(b+c)\cos(\psi_i-\psi_s)\right)^2 \quad (26)$$

$$I_{B_g}(\frac{\pi}{2},0,\psi,a,b,c,d,\psi_i,\psi_s)=f^2\sin(2\psi+\psi_i+\psi_s)^2 \quad (27)$$

For surface plane (100) Raman intensity has the following view:

$$I_{A_g}(\frac{\pi}{2},\frac{\pi}{2},\psi,a,b,c,d,\psi_i,\psi_s)=\frac{1}{4}\left((c-a)\cos(2\psi+\psi_i+\psi_s)+2d\sin(2\psi+\psi_i+\psi_s)+(a+c)\cos(\psi_i-\psi_s)\right)^2$$

(28)

$$I_{B_g}(\frac{\pi}{2},\frac{\pi}{2},\psi,a,b,c,d,\psi_i,\psi_s)=0 \quad (29)$$

By the fitting of experimental angular Raman intensity dependencies of the several CBZD vibrational modes we verified that peak at 1636cm^{-1} (νCC vibration) belongs to the A_g mode. We made this conclusion because fitting the same peak to Raman tensor which corresponds to B_g mode was not possible and produced inconsistent results. After the fitting procedure based on A_g mode Raman tensor (Supplementary Figure 27) we obtained the following values of the material parameters: a=0.43, b=0.06, c=0.45, d=−0.81.

Supplementary Note 9: Sapphire Raman tensor model development

Crystallographic structure of monocrystalline sapphire belongs to point group D_{3d} and space group D_{3d}^{6}. As a result we have the following irreducible representation for optical modes[12]:

$$\Gamma_{opt} = 2A_{1g} + 2A_{1u} + 3A_{2g} + 2A_{2u} + 5E_g + 4E_u, \tag{30}$$

And the following representation for the phonon modes:

$$\Gamma_{phonon} = A_{2u} + A_u. \tag{31}$$

Due to the fact that elementary cell has the center of inversion, the Raman active vibrations are not present in the IR spectra, and vice versa. As a result, we have seven modes, which are active in Raman spectra: two A_{1g} and five E_g modes (Supplementary Figure 30). Raman scattering tensors in the crystal coordinate frame have the following form[2]:

$$\Re_{A_{1g}} = \begin{pmatrix} a & 0 & 0 \\ 0 & a & 0 \\ 0 & 0 & a \end{pmatrix}, \quad \Re_{E_{g(x)}} = \begin{pmatrix} c & 0 & 0 \\ 0 & -c & d \\ 0 & d & 0 \end{pmatrix}, \quad \Re_{E_{g(y)}} = \begin{pmatrix} 0 & -c & -d \\ -c & 0 & 0 \\ -d & 0 & 0 \end{pmatrix}. \tag{32}$$

Here a, b, c, d – material parameters.

By the use of Supplementary Equation 3, Raman intensity of A_{1g} and E_g modes can be written as:

$$\begin{aligned} I_{A_{1g}}(\theta,\phi,\psi,\psi_i,\psi_s) = \frac{1}{64}((a-b)\cos(2\theta-2\psi-\psi_i-\psi_s)+(a-b)(\cos(2\theta+2\psi+\psi_i+\psi_s)+ \\ +\cos(2\theta+\psi_i-\psi_s)+\cos(2\theta-\psi_i+\psi_s)-2\cos(2\psi+\psi_i+\psi_s))+2(3a+b)\cos(\psi_i-\psi_s))^2 \end{aligned} \tag{33}$$

$$\begin{aligned} I_{E_{g(x)}}(\theta,\phi,\psi,\psi_i,\psi_s) = \Big(\sin(\psi_i)\Big(\sin(\phi)\Big(\sin(\psi)\cos(\psi)\cos(\psi_s)\Big(c\Big(\cos^2(\theta)+1\Big)\sin(\phi)+d\sin(2\theta)\Big)- \\ -\cos(\theta)\sin^2(\psi)\sin(\psi_s)(c\cos(\theta)\sin(\phi)+2d\sin(\theta))+c\cos^2(\psi)\sin(\psi_s)\sin(\phi)\Big)- \\ -\cos(\phi)\cos(2\psi+\psi_s)(2c\cos(\theta)\sin(\phi)+d\sin(\theta))-\frac{1}{4}c\cos^2(\phi)\Big(-4\cos^2(\theta)\sin^2(\psi)\sin(\psi_s)+ \\ +(\cos(2\theta)+3)\sin(2\psi)\cos(\psi_s)+4\cos^2(\psi)\sin(\psi_s)\Big)\Big)+ \\ +\cos(\psi_i)\Big(\cos(\psi_s)\Big(\sin(\psi)\Big(c\sin(\psi)\sin^2(\phi)-c\sin(\psi)\cos^2(\phi)-2d\sin(\theta)\cos(\psi)\cos(\phi)\Big)- \\ -2\cos(\theta)\cos(\psi)\sin(\phi)(2c\sin(\psi)\cos(\phi)+d\sin(\theta)\cos(\psi))+c\cos^2(\theta)\cos^2(\psi)\cos(2\phi)\Big)+ \\ +\sin(\psi_s)\Big(\sin(\psi)\cos(\psi)\sin(\phi)\Big(c\Big(\cos^2(\theta)+1\Big)\sin(\phi)+ \\ +d\sin(2\theta)\Big)-\cos(2\psi)\cos(\phi)(2c\cos(\theta)\sin(\phi)+d\sin(\theta))-\frac{1}{4}c(\cos(2\theta)+3)\sin(2\psi)\cos^2(\phi)\Big)\Big)\Big)^2 \end{aligned} \tag{34}$$

$$I_{E_{g(y)}}(\theta,\phi,\psi,\psi_i,\psi_s) = \Big(\sin(\psi_i)\Big(\cos(\psi_s)\Big(\cos(\theta)\Big(-c\cos(2\psi)\cos^2(\phi) + c\cos(2\psi)\sin^2(\phi) -$$
$$-2d\sin(\theta)\sin(\psi)\cos(\psi)\cos(\phi)\Big) + \sin(\phi)\Big(c\sin(2\psi)\cos(\phi) + d\sin(\theta)\sin^2(\psi) - d\sin(\theta)\cos^2(\psi)\Big) +$$
$$+2c\cos^2(\theta)\sin(\psi)\cos(\psi)\sin(\phi)\cos(\phi)\Big) + 2\sin(\psi_s)(\cos(\theta)\sin(\psi)\cos(\phi) + \cos(\psi)\sin(\phi))(\sin(\psi)(d\sin(\theta) -$$
$$-c\cos(\theta)\sin(\phi)) + c\cos(\psi)\cos(\phi))\Big) + \cos(\psi_i)\Big(\cos(\theta)\Big(c\sin^2(\phi)\sin(2\psi+\psi_s) - c\cos^2(\phi)\sin(2\psi+\psi_s) +$$
$$+2d\sin(\theta)\cos(\psi)\cos(\phi)\cos(\psi+\psi_s)\Big) + \sin(\phi)\Big(2c\sin^2(\psi)\cos(\psi_s)\cos(\phi) + c\sin(2\psi)\sin(\psi_s)\cos(\phi) +$$
$$+d\sin(\theta)\sin^2(\psi)\sin(\psi_s) - d\sin(\theta)\cos^2(\psi)\sin(\psi_s) - 2d\sin(\theta)\sin(\psi)\cos(\psi)\cos(\psi_s)\Big) -$$
$$-c\cos^2(\theta)\cos(\psi)\sin(2\phi)\cos(\psi+\psi_s)\Big)\Big)^2$$

(35)

The general-form Raman intensity for E_g mode:

$$I_{E_g}(\theta,\phi,\psi,\psi_i,\psi_s) = \frac{1}{2}I_{E_{g(x)}}(\theta,\phi,\psi,\psi_i,\psi_s) + \frac{1}{2}I_{E_{g(y)}}(\theta,\phi,\psi,\psi_i,\psi_s) \quad (36)$$

Using Supplementary Equation 32-36 it possible to obtain Raman intensity for different cuts of monocrystalline sapphire as a function of ψ , ψ_i , ψ_s .

<u>c-plane/a-axis</u> ($\theta = 0$, $\phi = 0$)

$$I_{A_{1g}}(0,0,\psi,\psi_i,\psi_s) = c^2\cos^2(\psi_i - \psi_s) \quad (37)$$

$$I_{E_g}(0,0,\psi,\psi_i,\psi_s) = \frac{c^2}{2} \quad (38)$$

<u>a-plane/c-axis</u> ($\theta = \frac{\pi}{2}$, $\phi = 0$)

$$I_{A_{1g}}(\frac{\pi}{2},0,\psi,\psi_i,\psi_s) = \frac{1}{4}((d-c)\cos(\psi_i + 2\psi + \psi_s) + (c+d)\cos(\psi_i - \psi_s))^2 \quad (39)$$

$$I_{E_g}(\frac{\pi}{2},0,\psi,\psi_i,\psi_s) = \frac{1}{8}(-c\cos(\psi_i + 2\psi + \psi_s) + c\cos(\psi_i - \psi_s) + 2d\sin(\psi_i + 2\psi + \psi_s))^2 \quad (40)$$

<u>m-plane/c-axis</u> ($\theta = \frac{\pi}{2}$, $\phi = \frac{\pi}{2}$)

$$I_{A_{1g}}(\frac{\pi}{2},\frac{\pi}{2},\psi,\psi_i,\psi_s) = \frac{1}{4}((d-c)\cos(\psi_i + 2\psi + \psi_s) + (c+d)\cos(\psi_i - \psi_s))^2 \quad (41)$$

$$I_{E_g}(\frac{\pi}{2},\frac{\pi}{2},\psi,\psi_i,\psi_s) = \frac{1}{16}(-2c^2\cos(2(\psi_i + \psi)) + c^2\cos(2(\psi_i + 2\psi + \psi_s)) + c^2\cos(2\psi_i - 2\psi_s) -$$
$$-2c^2\cos(2(\psi + \psi_s)) + 2c^2 - 4d^2\cos(2(\psi_i + 2\psi + \psi_s)) + 4d^2)$$

(42)

<u>r-plane/a-axis</u> ($\theta = \frac{58\pi}{180}$, $\phi = \frac{\pi}{2}$)

$$I_{A_{1g}}(\frac{58\pi}{180},\frac{\pi}{2},\psi,\psi_i,\psi_s) = ((0.36d - 0.36c)\cos(\psi_i + 2\psi + \psi_s) + (0.64c + 0.36d)\cos(\psi_i - \psi_s))^2$$

(43)

$$I_{E_g}(\frac{58\pi}{180},\frac{\pi}{2},\psi,\psi_i,\psi_s)=0.5\Big(((-0.64c-0.45d)\cos(\psi_i+2\psi+\psi_s)+(0.36c-0.45d)\cos(\psi_i-\psi_s))^2+$$
$$+(\cos(\psi_i)((0.53c-0.85d)\cos(2\psi)\sin(\psi_s)+(1.06c-1.7d)\sin(\psi)\cos(\psi)\cos(\psi_s))+$$
$$+(0.85d-0.53c)\sin(\psi_i)(2\sin(\psi)\cos(\psi)\sin(\psi_s)-\cos(2.\psi)\cos(\psi_s)))^2\Big) \quad (44)$$

Raman intensity of A_{1g} mode for $a-$ and $m-$planes are identical. As a result, it is better to use Raman intensity of E_g mode for the orientation determination.

Supplementary Note 10: Poly-sapphire 3D mapping

As an example of raw polarized Raman spectrum of monocrystalline sapphire we show spectrum acquired at r-plane in on-axis configuration $z(0\ 90)\bar{z}$ (Supplementary Figure 30). For the sapphire model development experiments we performed measurements in 2D-SAROM configuration (Supplementary Figure 8). System in 2D-SAROM configuration gave us an opportunity to collect non-overlapped polarized spectra of a-, c-, m- and r-planes at multiple polarization combinations and fit intensity versus rotation dependencies using Supplementary Equation 3.

By the analysis of obtained data in 2D-SAROM configuration, we verified that polarized Raman spectra of sapphire are rather similar for a- and r- planes. Among two A_{1g} modes (417 and 645cm^{-1}) and five E_g modes (378, 430, 451, 578 and 750cm^{-1}) of sapphire[2] we chose fifth E_g mode (750cm^{-1}) for the further analysis because it provides more variation in polarized Raman spectra than other modes where a- and r-planes were successfully distinguished (Supplementary Figure 29). Raman tensor element values obtained by fitting are c=0.045, d=−0.49 which are rather close to the values presented in reference[2].

An example of 3D plots of the Raman spectra of sapphire for polarization configuration $z(0\ 90)\bar{z}$ versus sample rotation angle ψ for a-, c-, m- and r-planes is shown in Supplementary Figure 29. Comparison between theoretical (red line) and experimental (blue circles) responses of the $E_g(5)$ mode versus sapphire sample rotation angle ψ plotted in polar coordinates for polarization configuration $z(0\ 90)\bar{z}$ is shown in Supplementary Figure 31.

In order to keep comparable signal to noise ratio of Raman spectra captured at different depth during 3D mapping of the sample, we implemented depth-dependent acquisition algorithm which exponentially increases exposure time for each mapping Z stack. Therefore, polarized 3D measurements of polycrystalline sapphire were performed with exposure time per point varied from 50ms to 2s resulting in total mapping time 176min. Due to the aberration corrected SAROM system design, we were able to reach a diffraction limited axial resolution without a pinhole which is used in classical confocal microscopy schemes. The confocal mode was organized in a cross slit geometry, where the vertical slit orientation is implemented at the entrance of the spectrometer and the horizontal slit orientation is performed by the readout of the selected rows on the spectroscopic CCD focal plane (Supplementary Figure 37).

We performed a simulation of lateral (Supplementary Figure 36f, 36g) and axial (Supplementary Figure 36d, 36e, 36h, 36i) resolution for typically used high resolution dry metallurgic objective in confocal Raman microscopy (Supplementary Figure 36a) and self-designed oil immersion objective for 3D measurements in Raman microscopy (Supplementary Figure 36b). For the correct

comparison, as a dry objective, we have chosen an infinity-corrected, semi-apochromatic microscope objective having an N.A. of 0.85 at laser beam diameter 6.5mm which provides a magnification of 50 times when used with a telescope objective having an effective focal length of 183 mm (Olympus patent US04417787) (Supplementary Figure 36a). Our self-designed objective has similar parameters: NA=0.85 at laser beam diameter of 8.8mm, magnification 45x at telescope objective having an effective focal length of 183 mm (Supplementary Figure 36b).
Therefore, both objectives provided equal lateral resolution on the sample surface (black lines in Supplementary Figure 36f, 36g). However, Huygens point spread function (PSF) simulation has shown that axial resolution becomes significantly worth in the case of dry objective in the depth of sapphire (-50µm) (Supplementary Figure 36h) rather than for oil immersion objective (Supplementary Figure 36i). Taking into account the values on the scale bar in Supplementary Figure 36d-36i, it is possible to conclude that aberration driven signal attenuation in the depth of 50µm is around 55% for dry metallurgic objective and 11% for oil immersion objective.
During 3D mapping of sapphire using oil immersion objective (Supplementary Figure 36b) we observed much more signal attenuation than 11% due to signal absorbance and reflections on grains in polycrystalline sapphire.
Polycrystalline sapphire manifested fluorescence at the grain boundaries, a perfect mark for grain visualization. In this particular sample we decided to realize grain segmentation based on the combined response from fluorescence and Raman channels. Fluorescence-based signal helped us to avoid ambiguities in the determination of grain boundaries which could be present when Raman signal is used (for example, when neighboring domains have identical or close orientation). However, the usage of only Raman maps obtained at different polarization configurations for segmentation is also a good decision in the general case. A comparison of segmentation results obtained on fluorescence and Raman responses is shown in Supplementary Figure 32. The illustration of segmentation procedure applied to the nine acquired polarized Raman maps at first 2D layer in 3D map presented in Supplementary Figure 33, where grain boundary distribution map based on the fluorescence peak intensity at 1418 cm^{-1} shown on top, segmented domains based on combined fluorescence and Raman responses are shown under it. Described segmentation was applied to each layer in 3D (Supplementary Figure 34).
3D orientation map of polycrystalline sapphire was visualized in Paraview software.

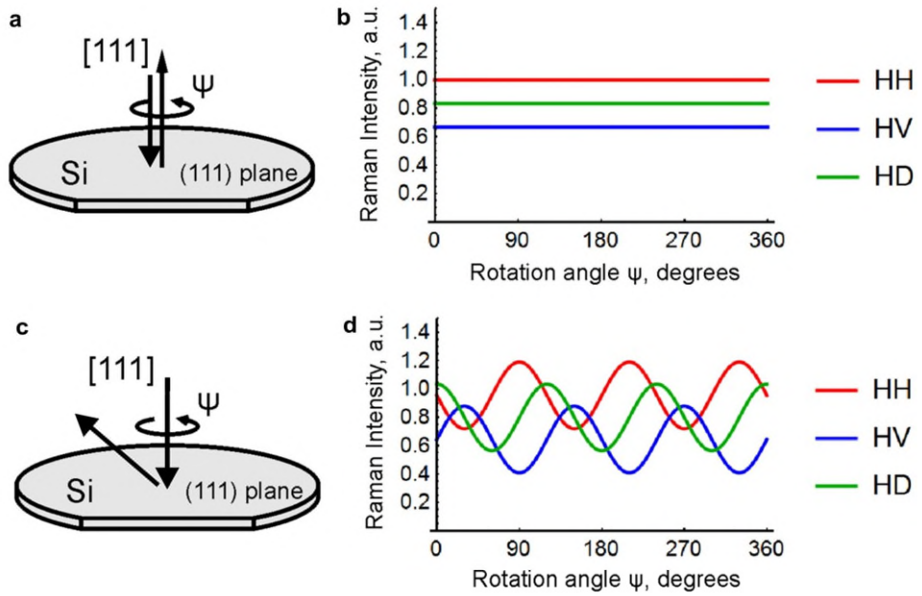

Supplementary Figure 1. On axis and off-axis registration differences. a, On-axis scheme with normal sample illumination and on-axis collection of scattered light and **b,** corresponding Raman intensity dependence on wafer rotation angle ψ, **c,** off-axis illumination scheme and **d,** Raman intensity dependence on wafer rotation angle ψ. HH – horizontal/horizontal polarizer/analyzer configuration, HV – horizontal/vertical polarizer/analyzer configuration, HD - horizontal/tilted at 45 degrees polarizer/analyzer configuration.

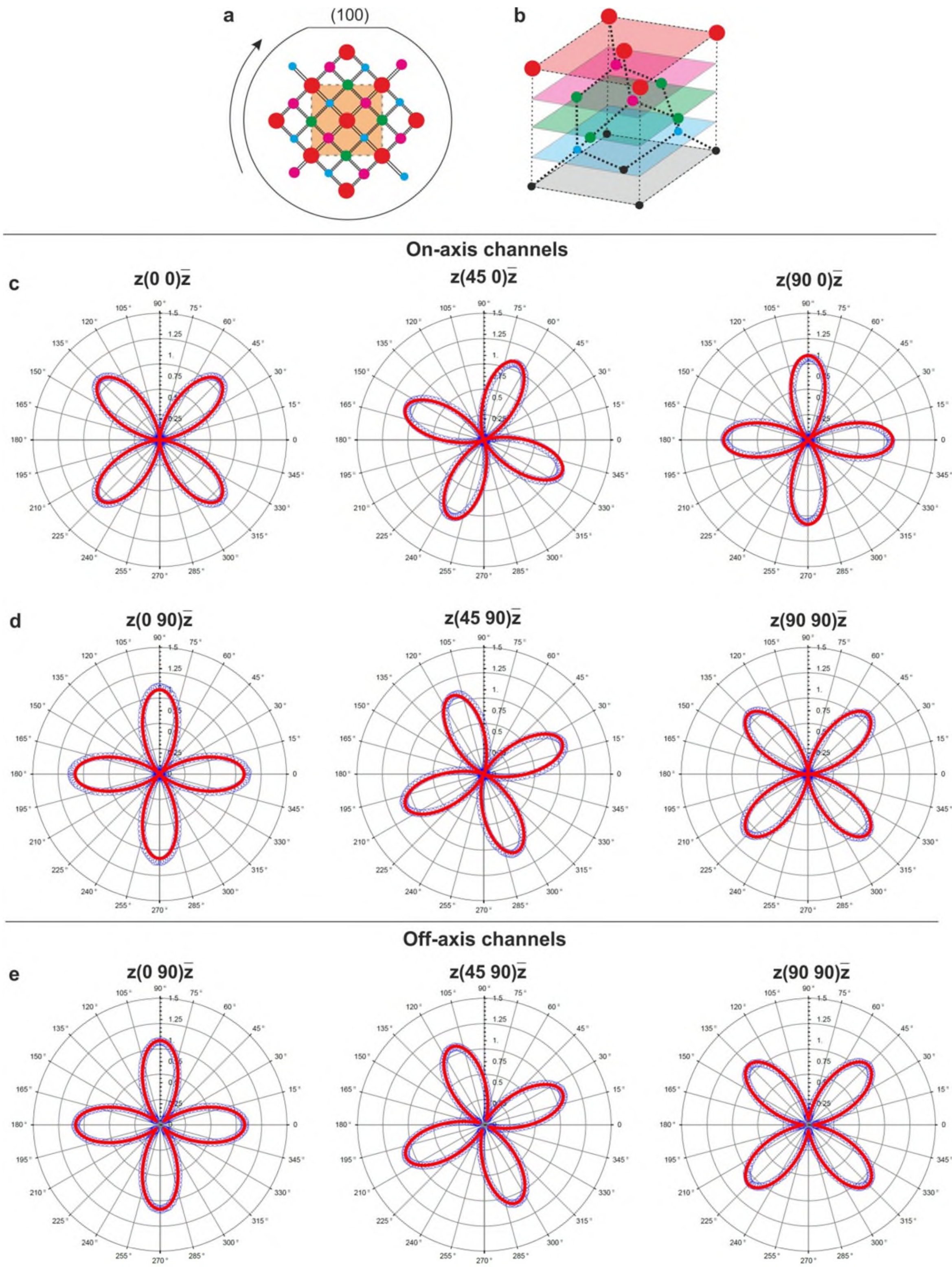

Supplementary Figure 2. Wafer rotation experiment on Si (100). a,b, Top and 3D view of Si unit cell. Comparison between theoretical (red line) and experimental (blue circles) responses of the sum of Si modes versus wafer rotation angle ψ plotted in polar coordinates for Si wafer with surface plane (100) for on-axis (**c,d)** and off-axis (**e)** polarization channels of SAROM system.

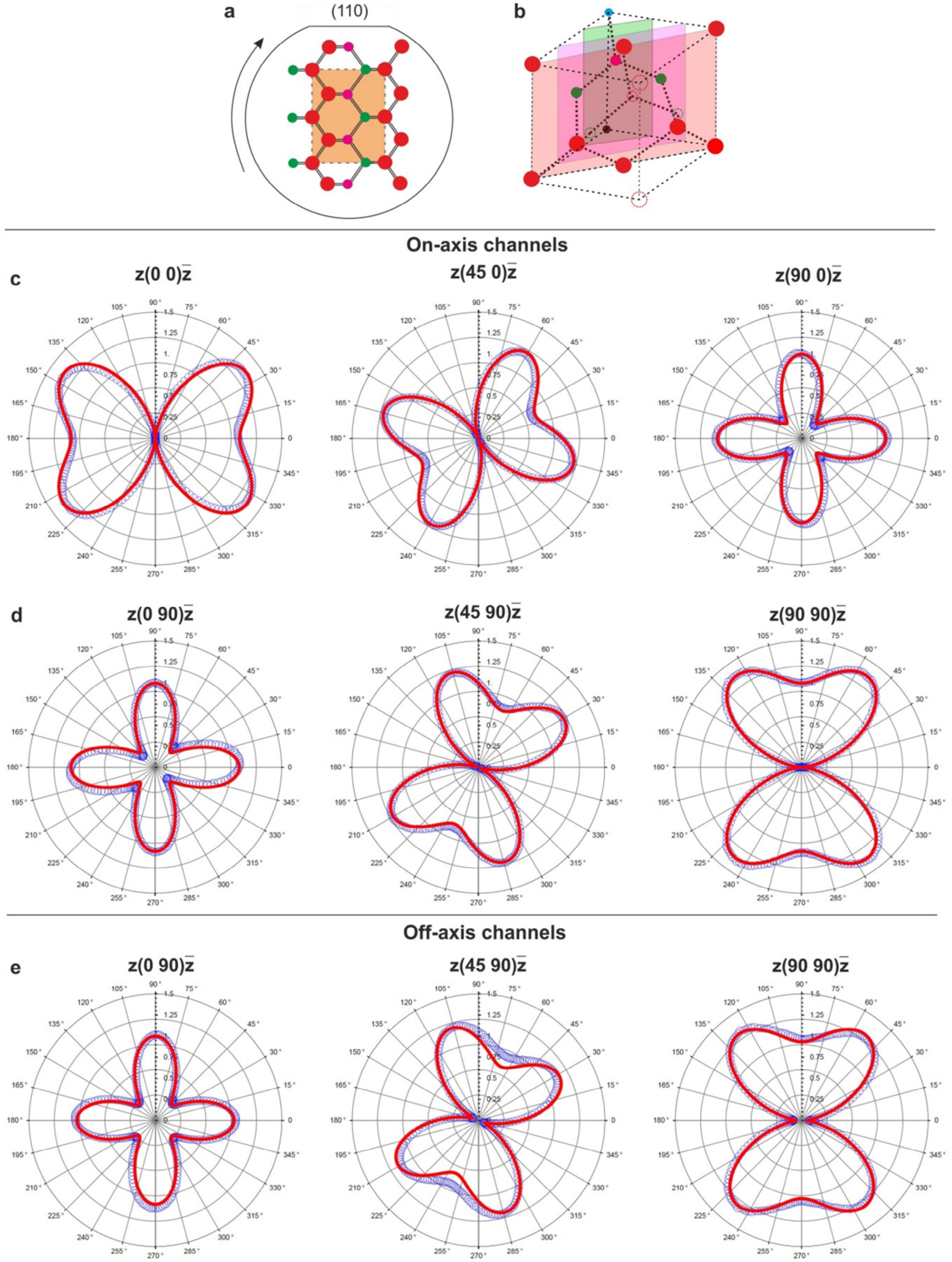

Supplementary Figure 3. Wafer rotation experiment on Si (110). a,b, Top and 3D view of Si unit cell. Comparison between theoretical (red line) and experimental (blue circles) responses of the sum of Si modes versus wafer rotation angle ψ plotted in polar coordinates for Si wafer with surface plane (110) for on-axis (**c,d)** and off-axis (**e)** polarization channels of SAROM system.

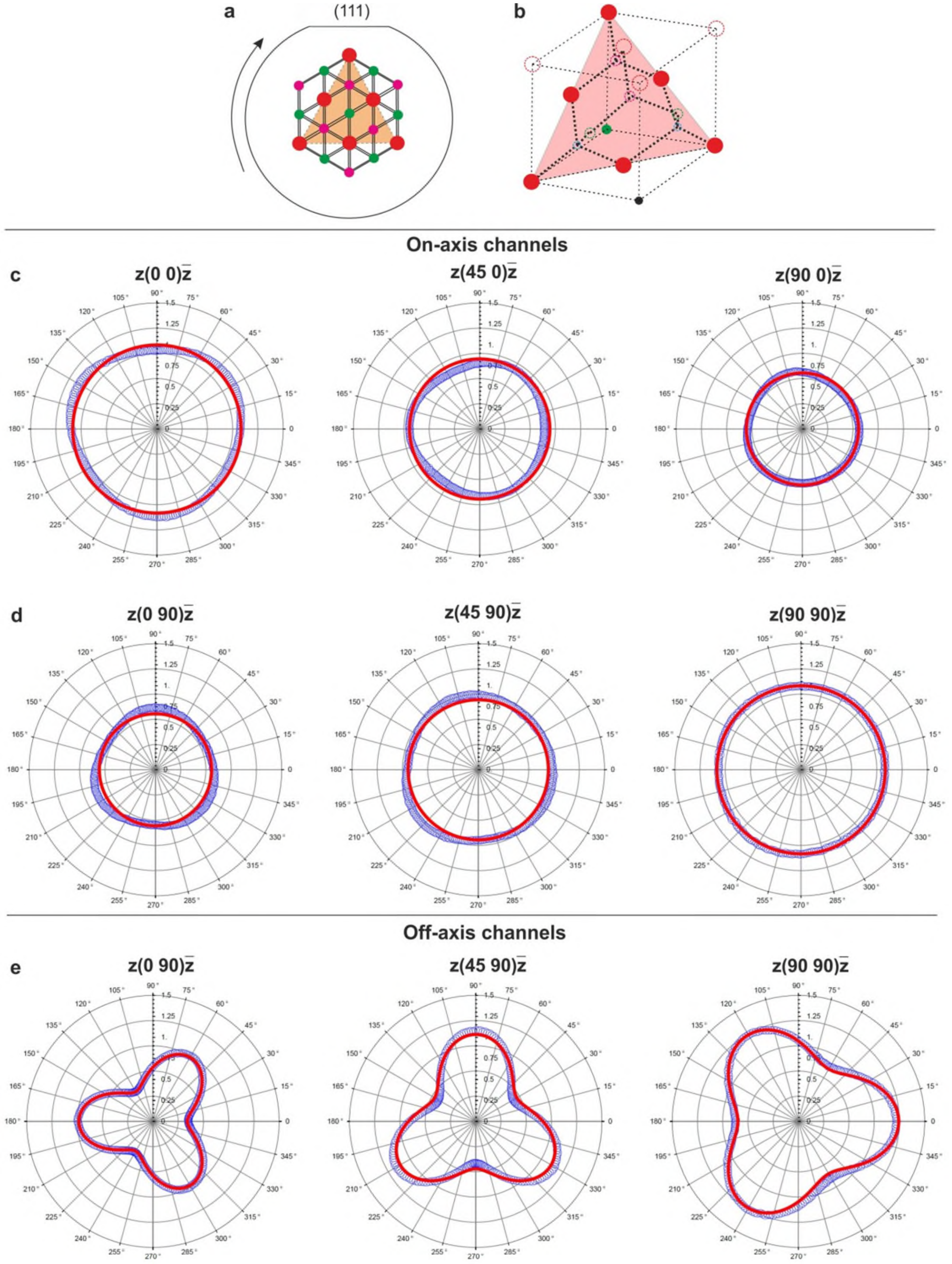

Supplementary Figure 4. Wafer rotation experiment on Si (111). a,b, Top and 3D view of Si unit cell. Comparison between theoretical (red line) and experimental (blue circles) responses of the sum of Si modes versus wafer rotation angle ψ plotted in polar coordinates for Si wafer with surface plane (111) for on-axis (**c,d**) and off-axis (**e**) polarization channels of SAROM system.

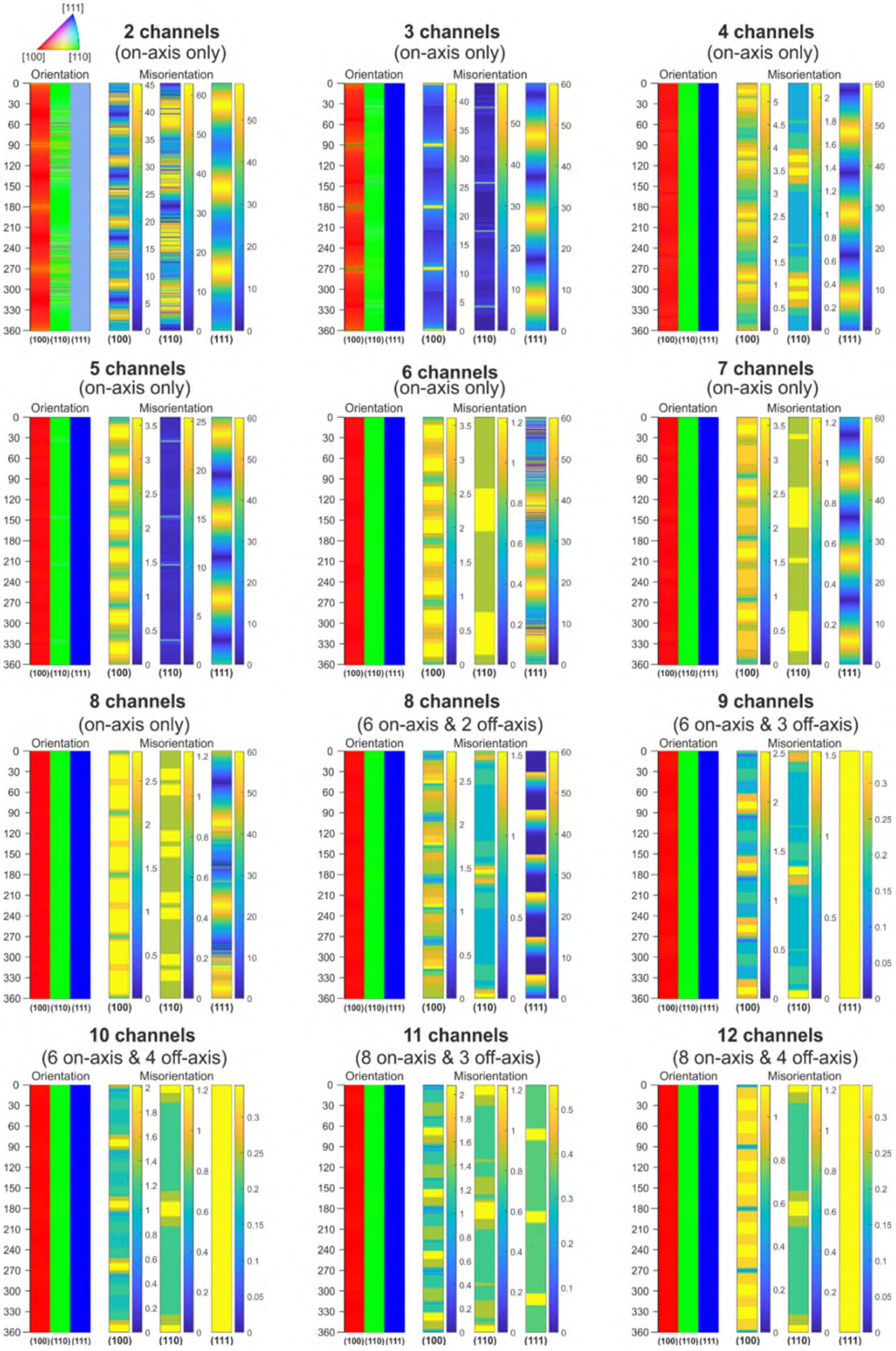

Supplementary Figure 5. Simulation based orientation determination error versus a number of polarized channels. The colors refer to the orientations shown by the inverse pole figure (inserted). Misorientation data presented separately on each set. Refer to colored scale bars for misorientation angle range.

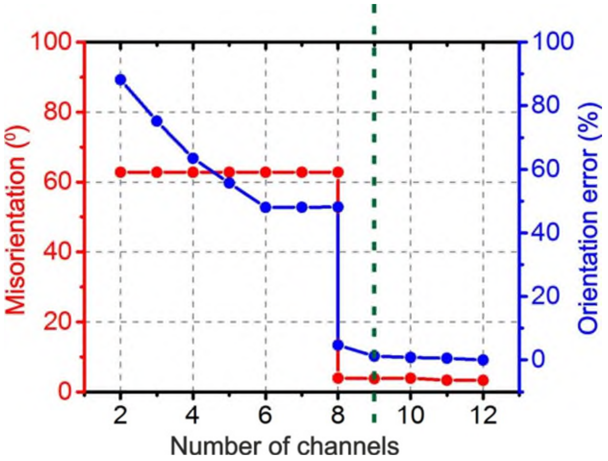

Supplementary Figure 6. Simulation based orientation determination error and misorientation versus the number of polarized channels.

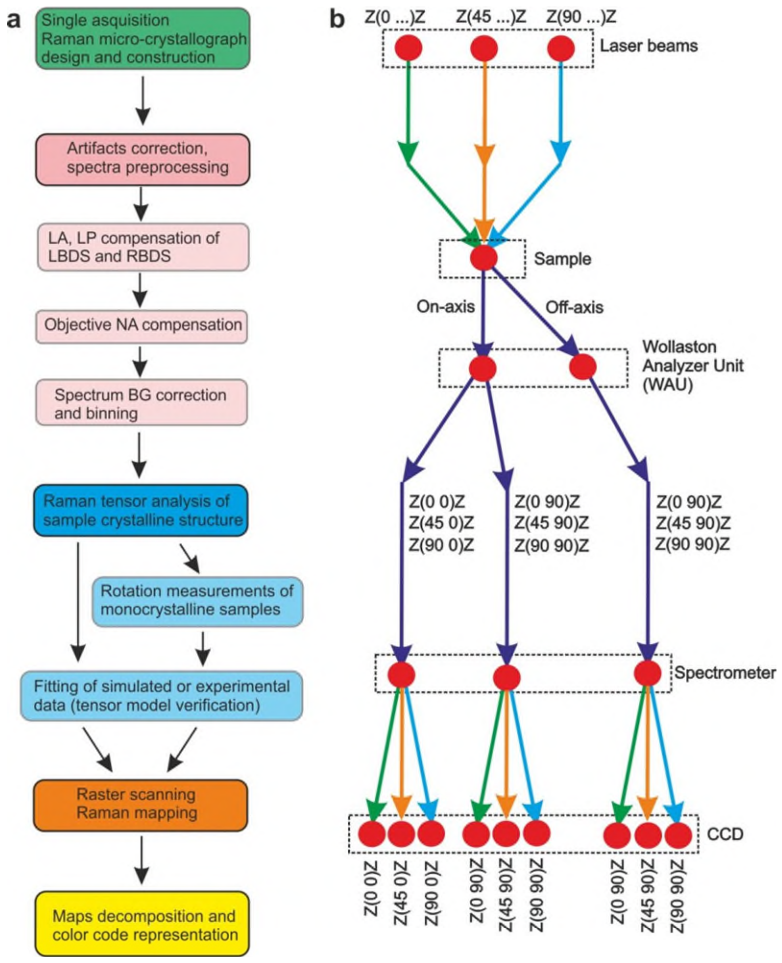

Supplementary Figure 7. SAROM principle. Illustration of a SAROM workflow (**a)** and schematic representation of simultaneous measurements with nine polarized Raman channels (**b)**.

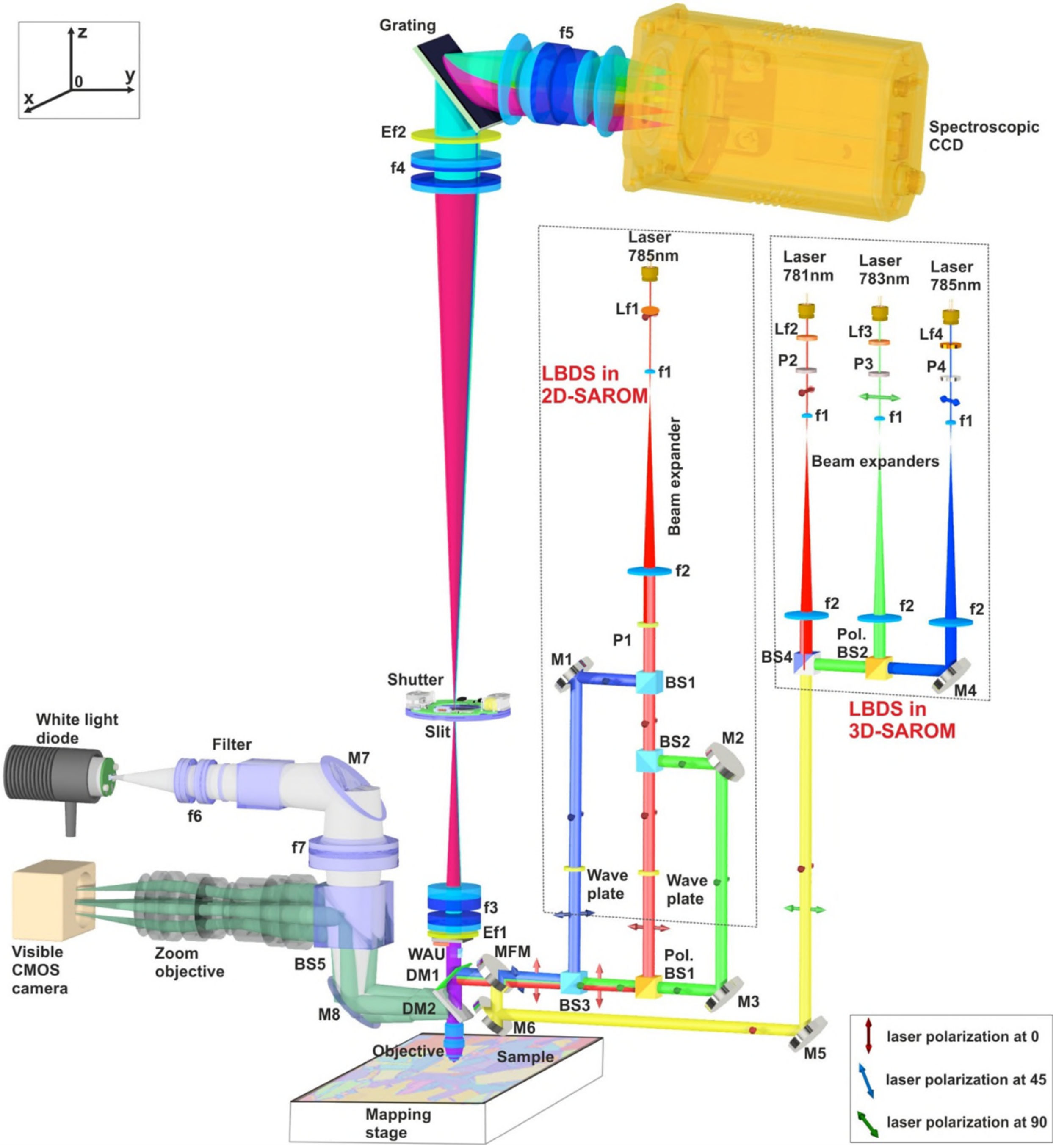

Supplementary Figure 8. Detailed illustration of SAROM optical design. Abbreviations: LBDS – Laser Beam Delivery System, Lf – laser filter, P – polarizer, f – lens, BS – beam splitter, Pol.BS – polarized beam splitter, M – mirror, MFM – motorized flip mirror, DM – dichroic mirror, WAU – Wollaston Analyzer Unit, Ef – edge filter.

Unique properties of spectrograph design covered by pending patent of Technical University of Denmark (application number 18183474.8).

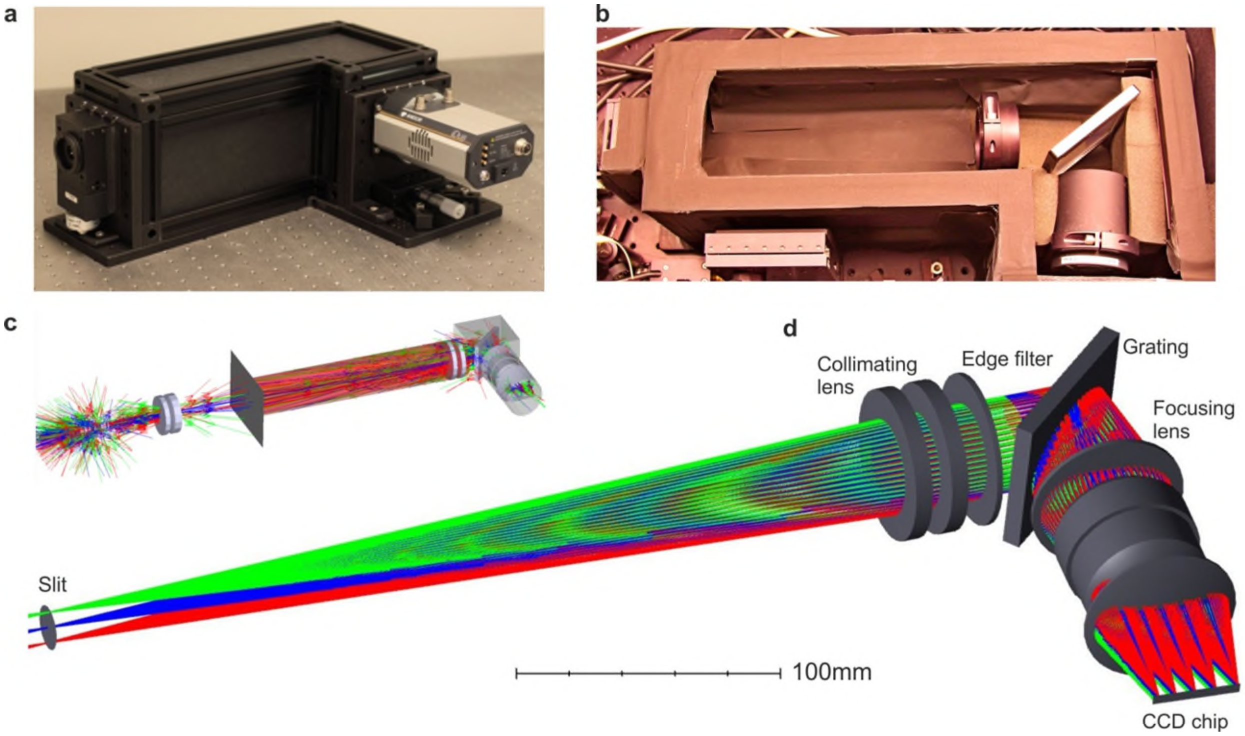

Supplementary Figure 9. SAROM spectrograph. a, Photograph of the spectrograph coupled with spectroscopic CCD, **b,** photograph of the spectrograph optical components, **c,** stray light analysis, **d,** optical design of the spectrograph. Unique features of spectrograph design are covered by pending patent of Technical University of Denmark (application number PCT/DK2019/050027).

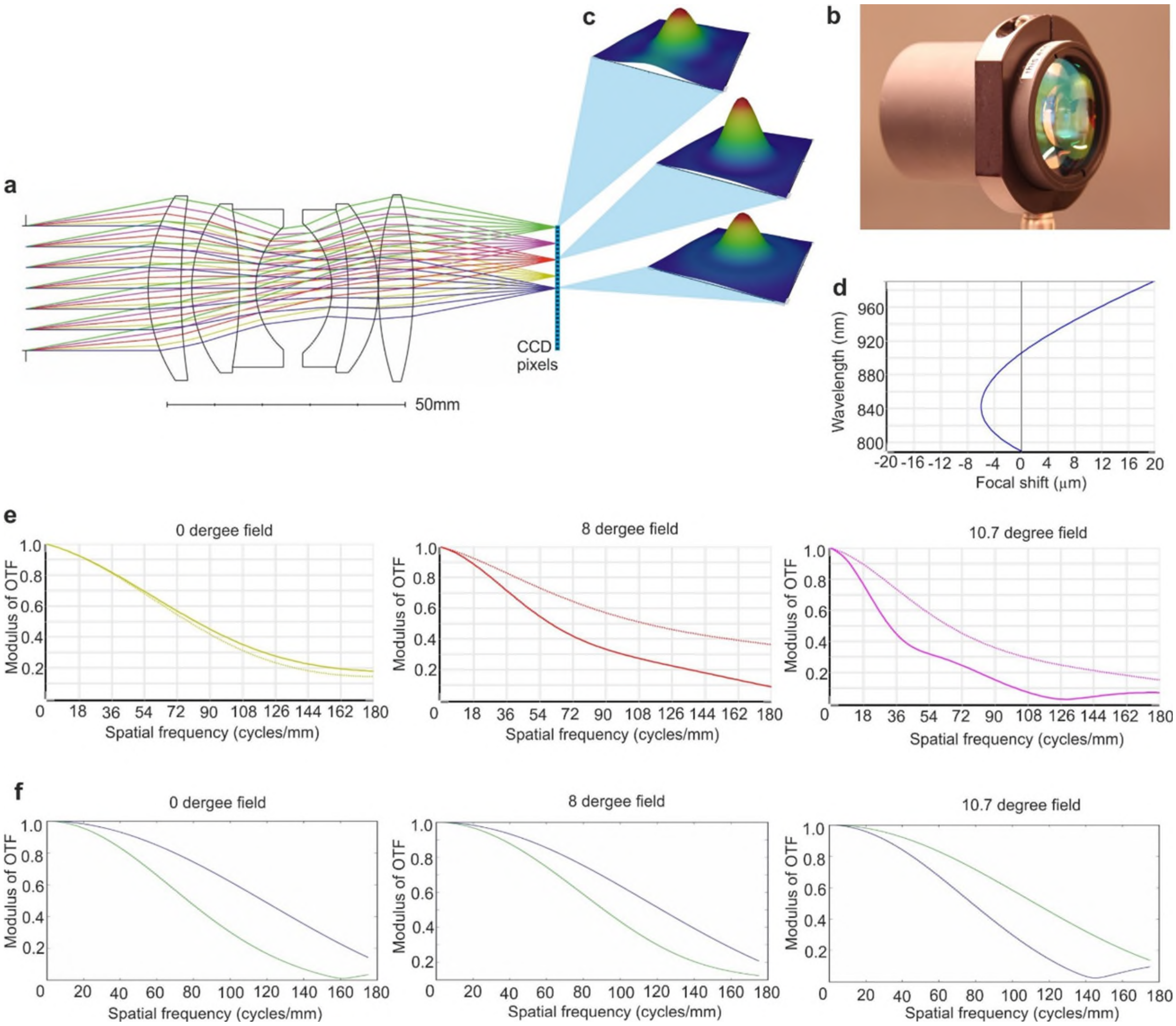

Supplementary Figure 10. Spectrograph focusing objective. a, optical design, **b,** photograph, **c,** point spread functions at different fields (0, 8 and 10.7 degrees), **d,** achromatic focal shift, experimental (**e)** and designed (**d)** modulus of OTF versus spatial frequency for different fields (0, 8 and 10.7 degrees).

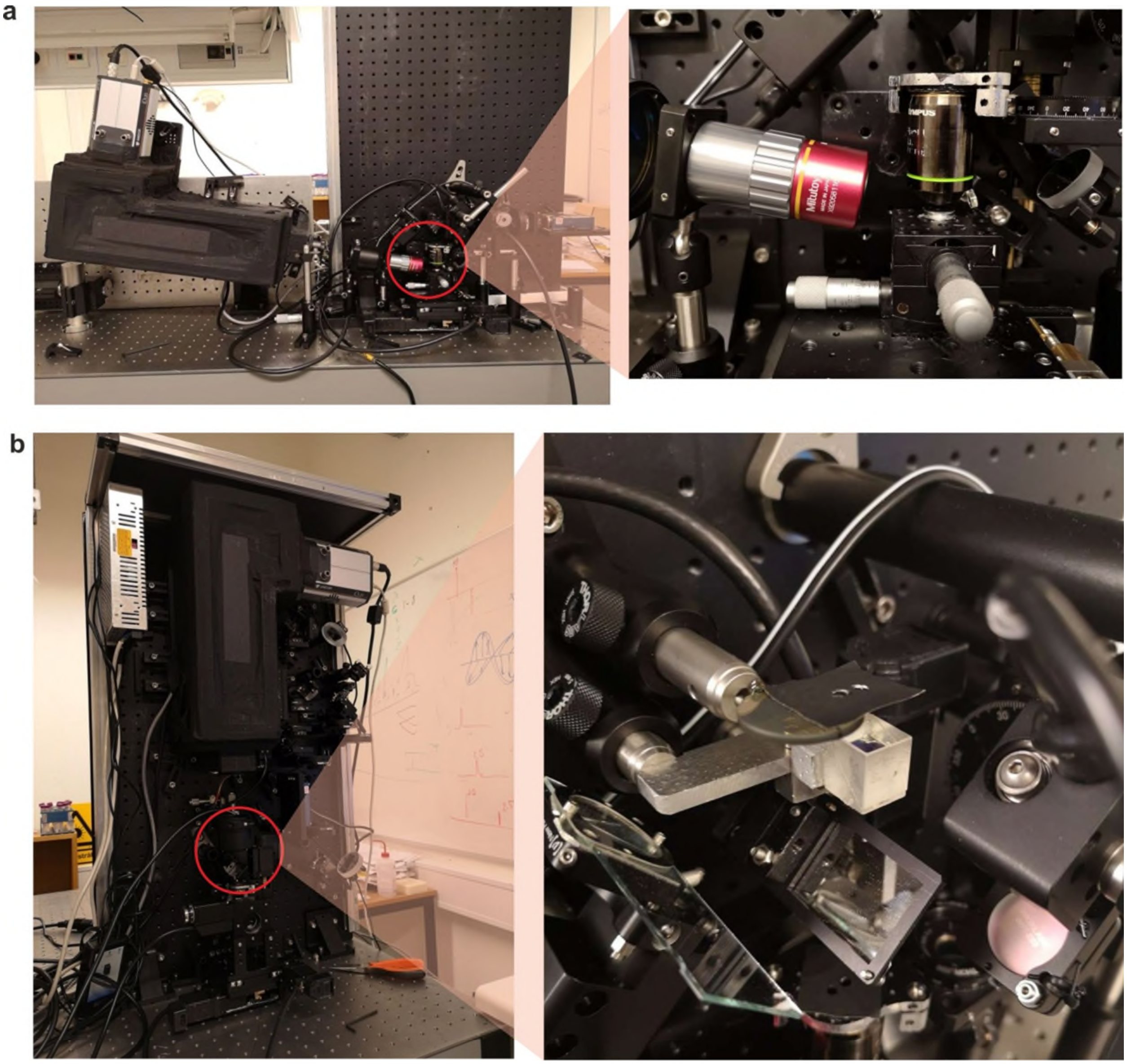

Supplementary Figure 11. Experimental setup development process. a, Photograph of setup for off-axis polarization experiments only, **b,** photograph of final SAROM setup with simultaneous on-axis and off-axis polarization measurements

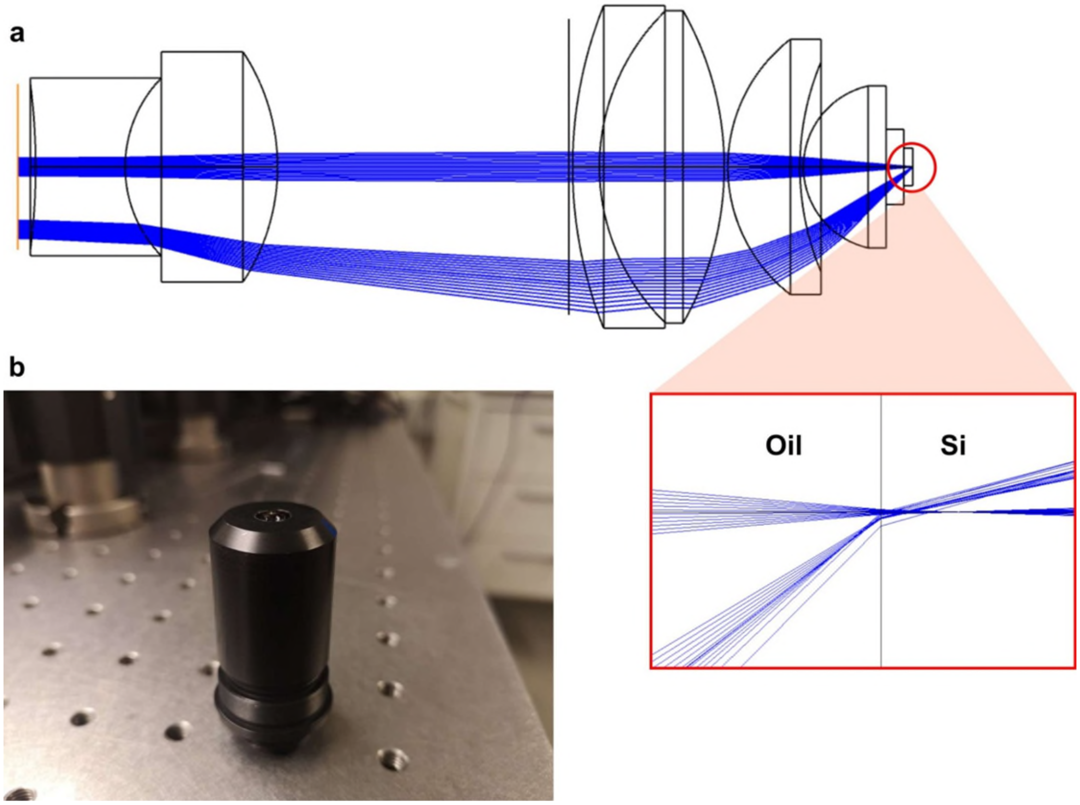

Supplementary Figure 12. Custom designed microscope objective. a, optical design of the objective (zoomed area illustrates the difference between collecting cones of light scattered in the Silicon sample at on-axis and off-axis geometries), **b,** objective photograph.

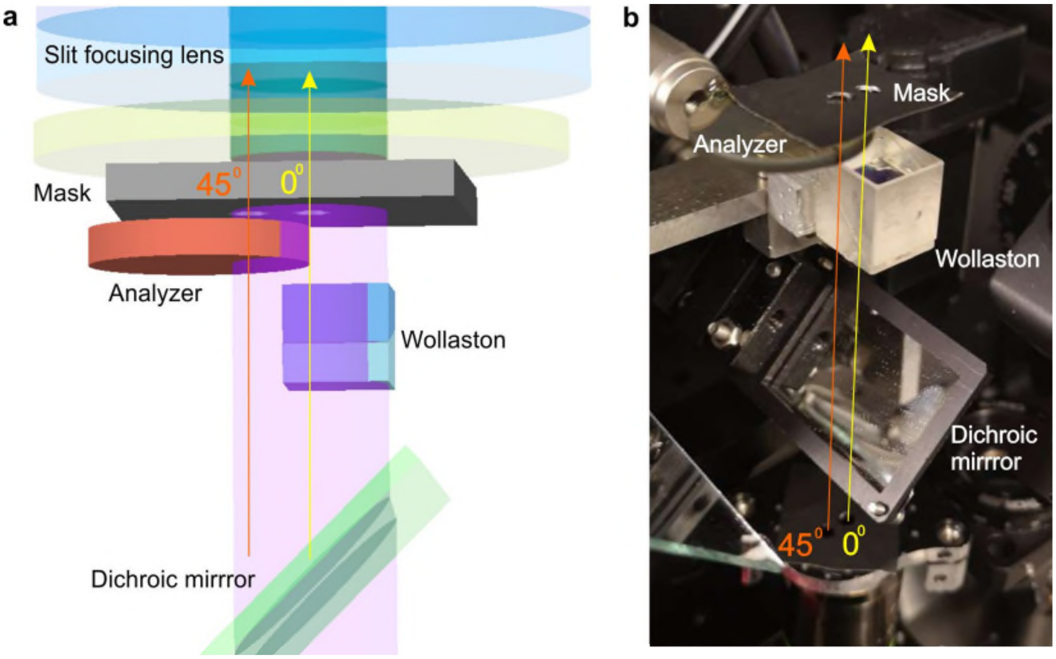

Supplementary Figure 13. Wollaston Analyzer Unit (WAU). a, Illustration of the Raman scattering beam path through WAU, **b,** photograph of WAU inserted into experimental setup.

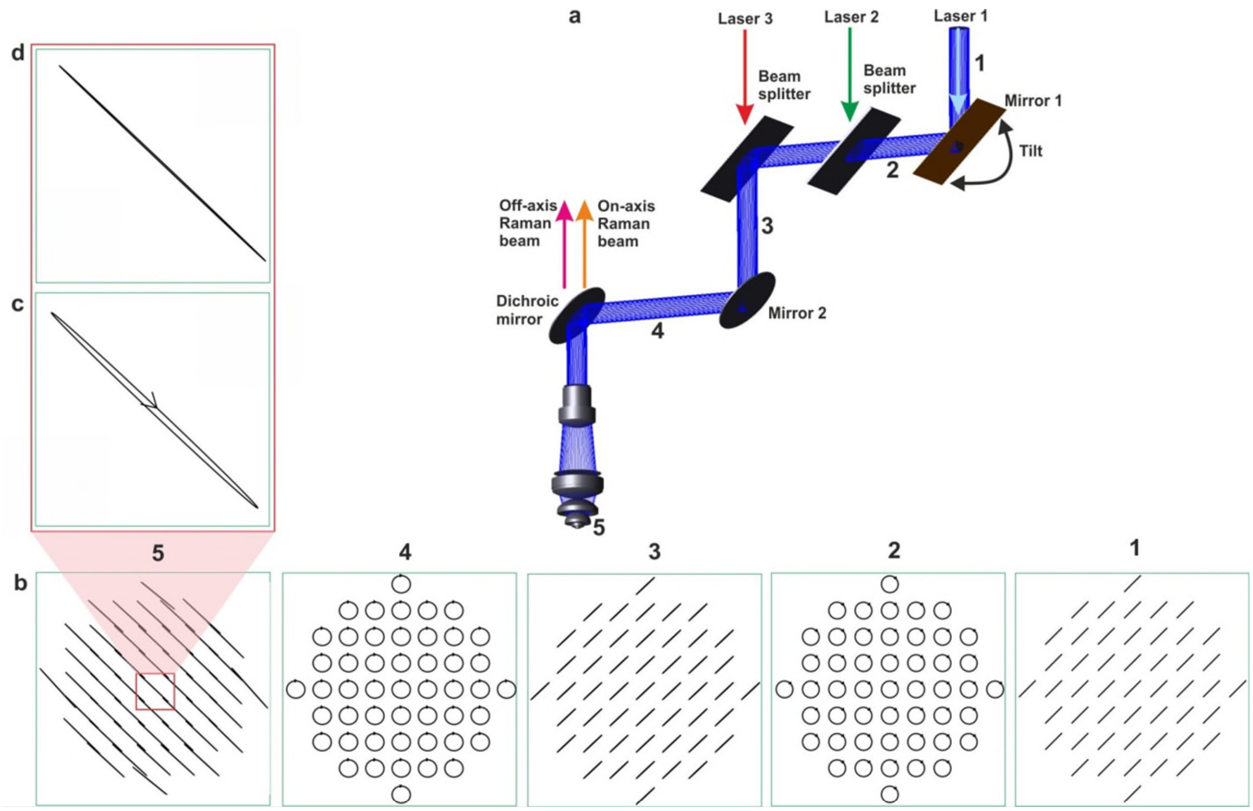

Supplementary Figure 14. Laser polarization propagation. a, Optical schema of Laser Beam Delivery System (LBDS), **b,** LBDS analyzed on laser polarization propagation with input angle of polarization state of 45°. The polarization state of laser beam focused onto diffraction limited spot on a sample focal plane before (**c**) and after (**d**), compensation of phase shift between S and P components of electromagnetic wave. Compensation was realized by the tilt of mirror M1 on 0.8°.

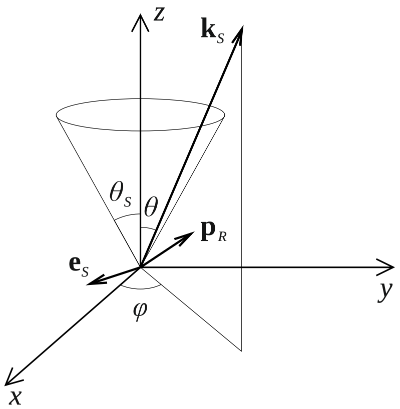

Supplementary Figure 15. Local spherical coordinate system associated with collection aperture.

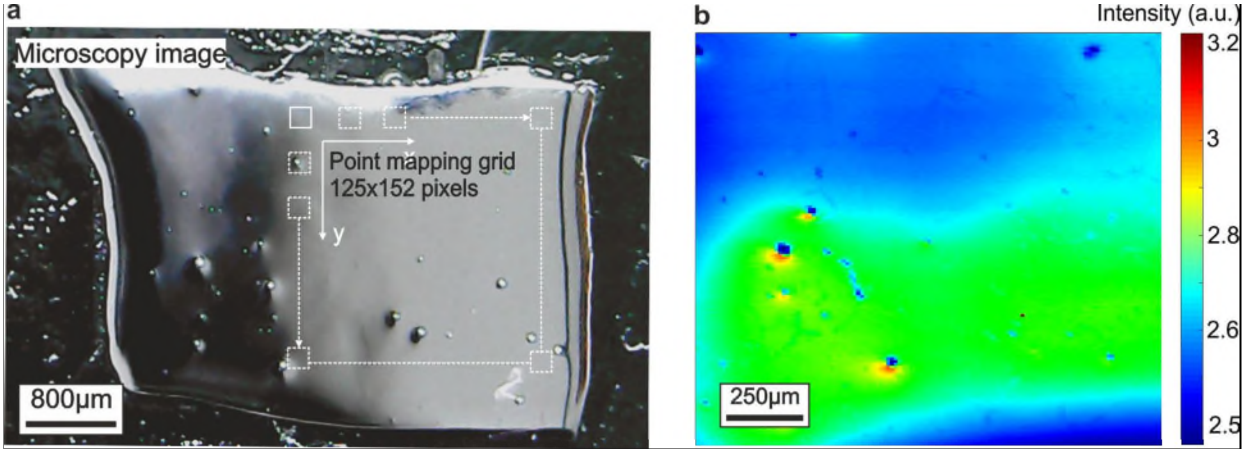

Supplementary Figure 16. Visible light and fluorescence microscopy of polycrystalline Si. a, Light microscopy image of the sample of polycrystalline Si, **b,** map of the surface of the polycrystalline Si plotted at the maximum of fluorescence intensity band.

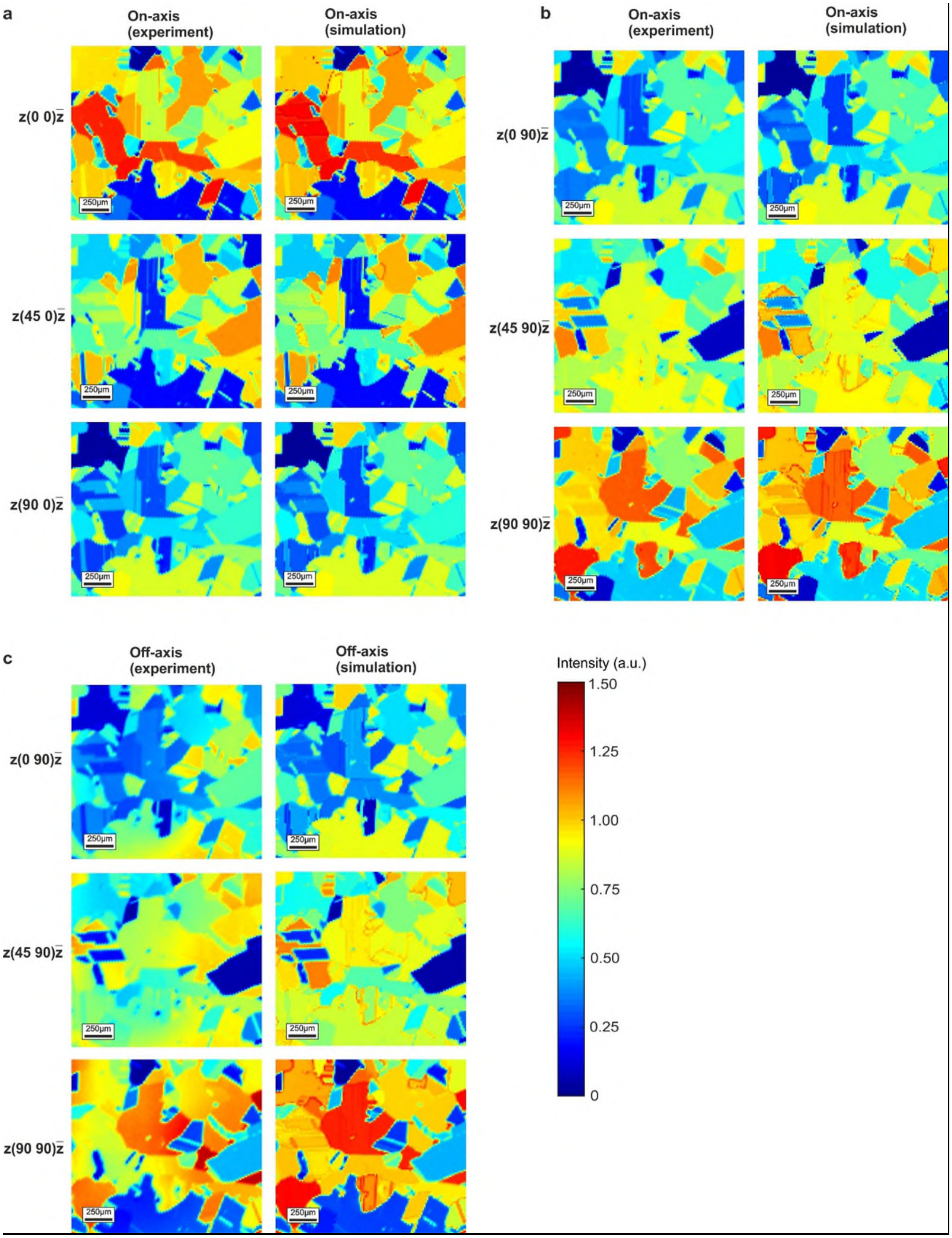

Supplementary Figure 17. Raman intensity map of polycrystalline Si. a, On-axis simulated and experimental Raman intensity maps at polarization channels $z(0\ 0)\bar{z}$, $z(45\ 0)\bar{z}$, $z(90\ 0)\bar{z}$, **b,**

on-axis simulated and experimental Raman intensity maps at polarization channels $z(0\ 90)\bar{z}$, $z(45\ 90)\bar{z}$, $z(0\,90)\bar{z}$, **c,** off-axis simulated and experimental Raman intensity maps at polarization channels $z(0\ 90)\bar{z}$, $z(45\ 90)\bar{z}$, $z(0\,90)\bar{z}$.

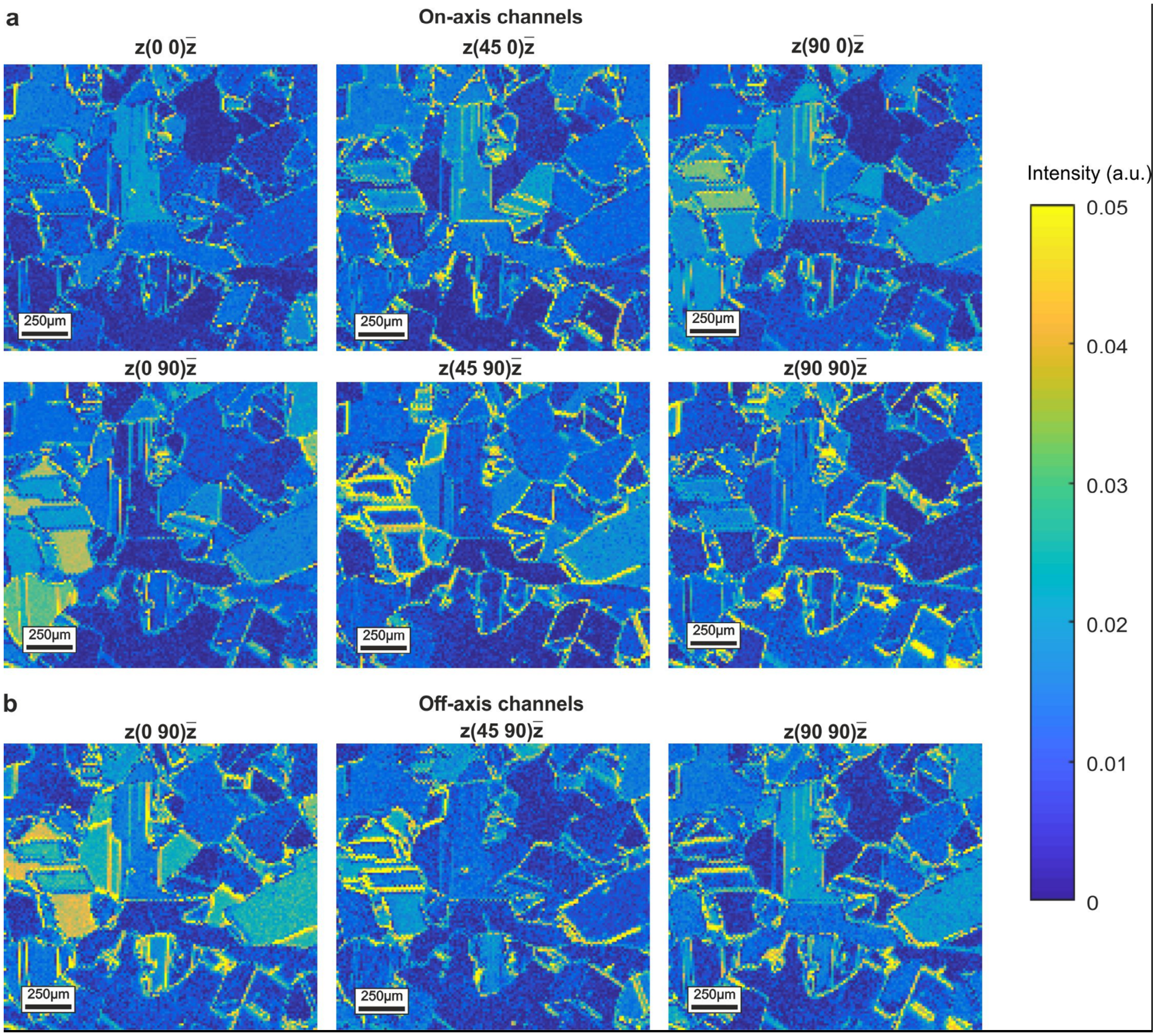

Supplementary Figure 18. Residual Raman intensity maps. **a,** On-axis residual Raman intensity maps at polarization channels $z(0\ 0)\bar{z}$, $z(45\ 0)\bar{z}$, $z(90\ 0)\bar{z}$, $z(0\ 90)\bar{z}$, $z(45\ 90)\bar{z}$ and $z(0\,90)\bar{z}$, **b,** off-axis residual Raman intensity maps at polarization channels $z(0\ 90)\bar{z}$, $z(45\ 90)\bar{z}$, $z(0\,90)\bar{z}$. Residual Raman intensity maps were obtained as a result of subtraction between simulated and experimental polarization data from each of the nine polarized channels.

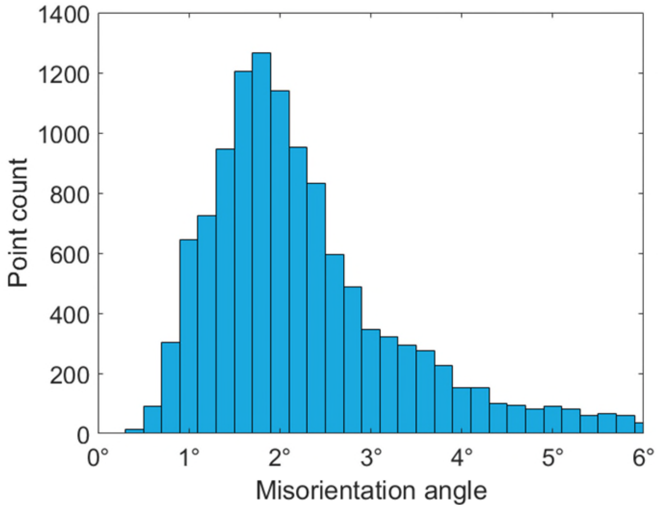

Supplementary Figure 19. Histogram of the misorientation map from the sample of polycrystalline Si.

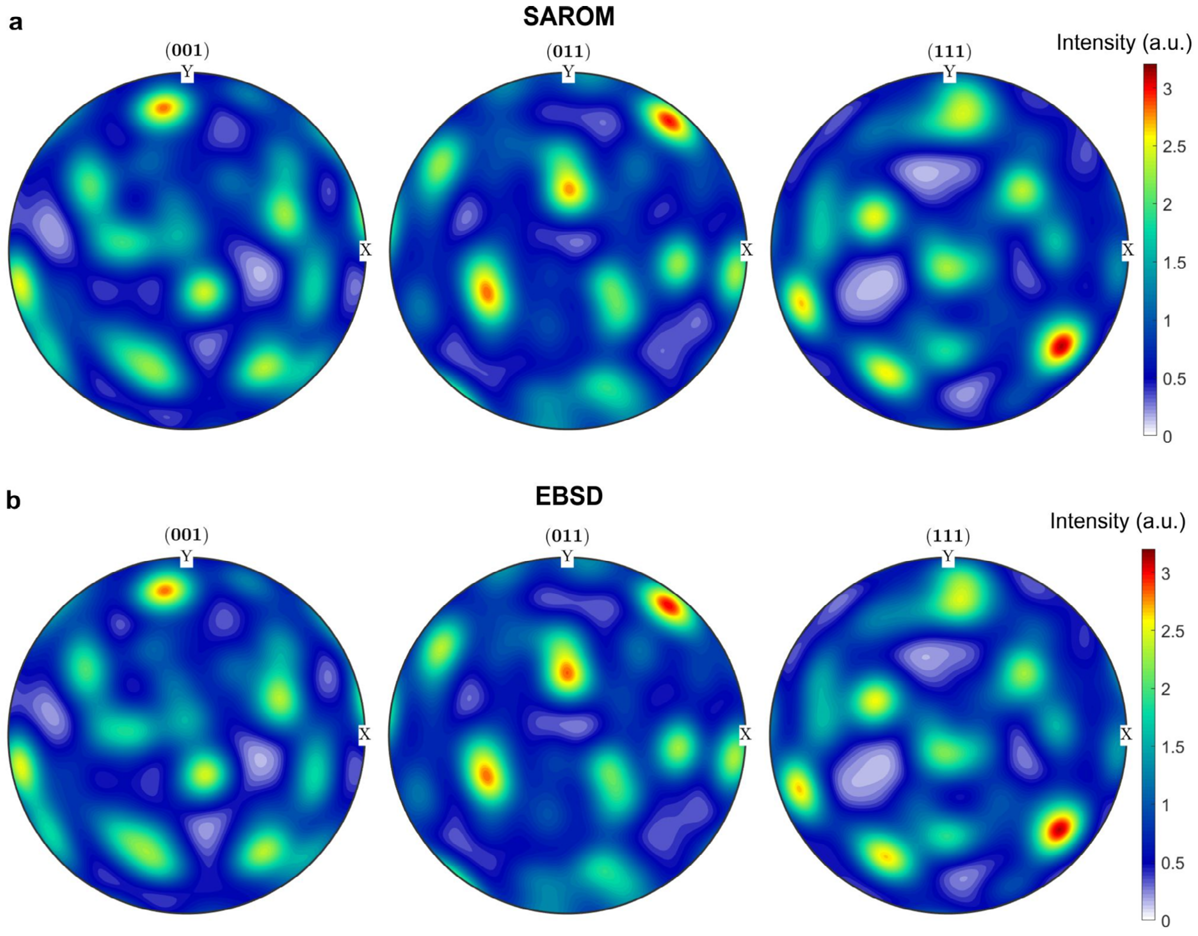

Supplementary Figure 20. Inverse Pole Figures of polycrystalline Si. Comparison between Inverse Pole Figures (IPF) plotted on SAROM (**a**) and EBSD (**b**) orientation maps of polycrystalline Si.

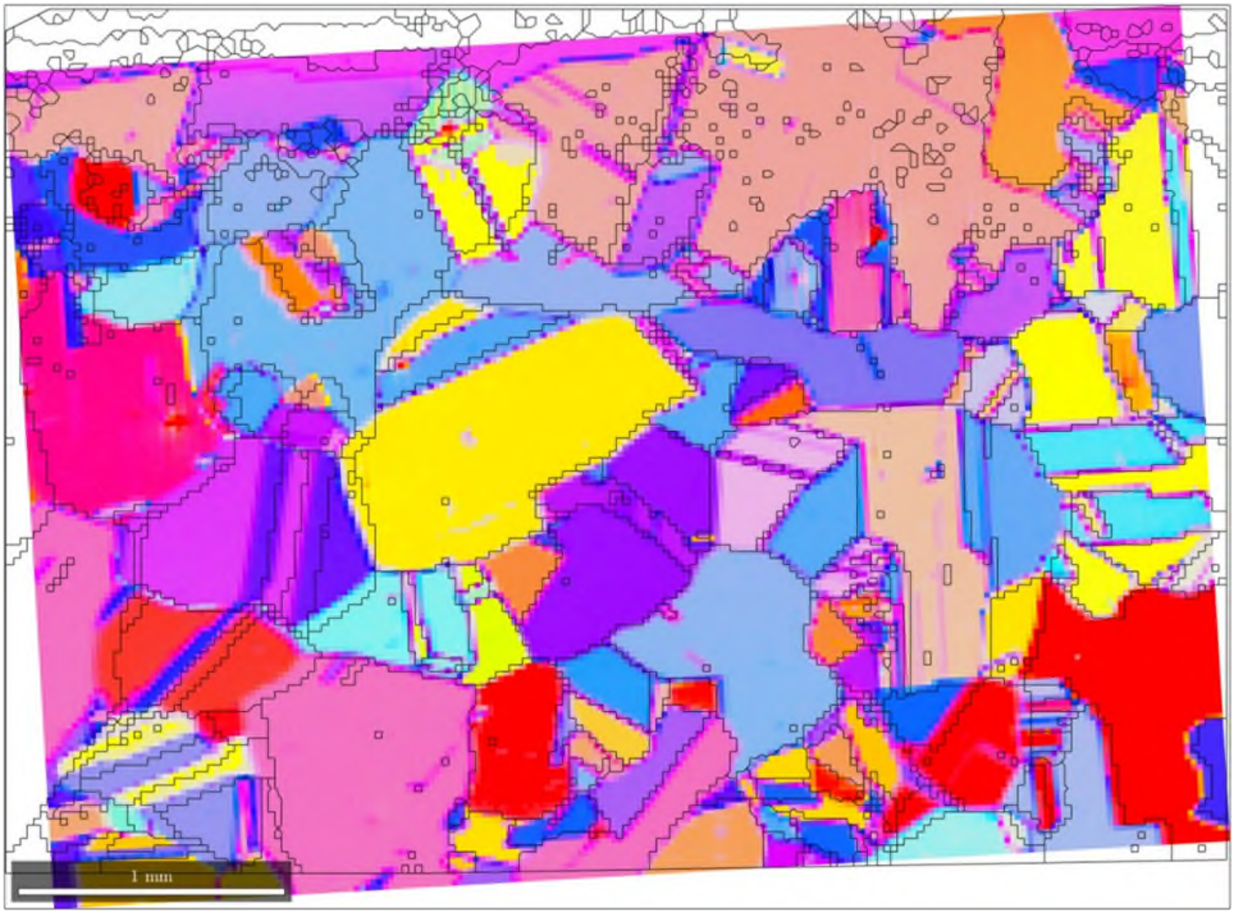

Supplementary Figure 21. Geometrical distortions. Illustration of the geometrical distortions of the EBSD map demonstrated on overlapping SAROM and EBSD images.

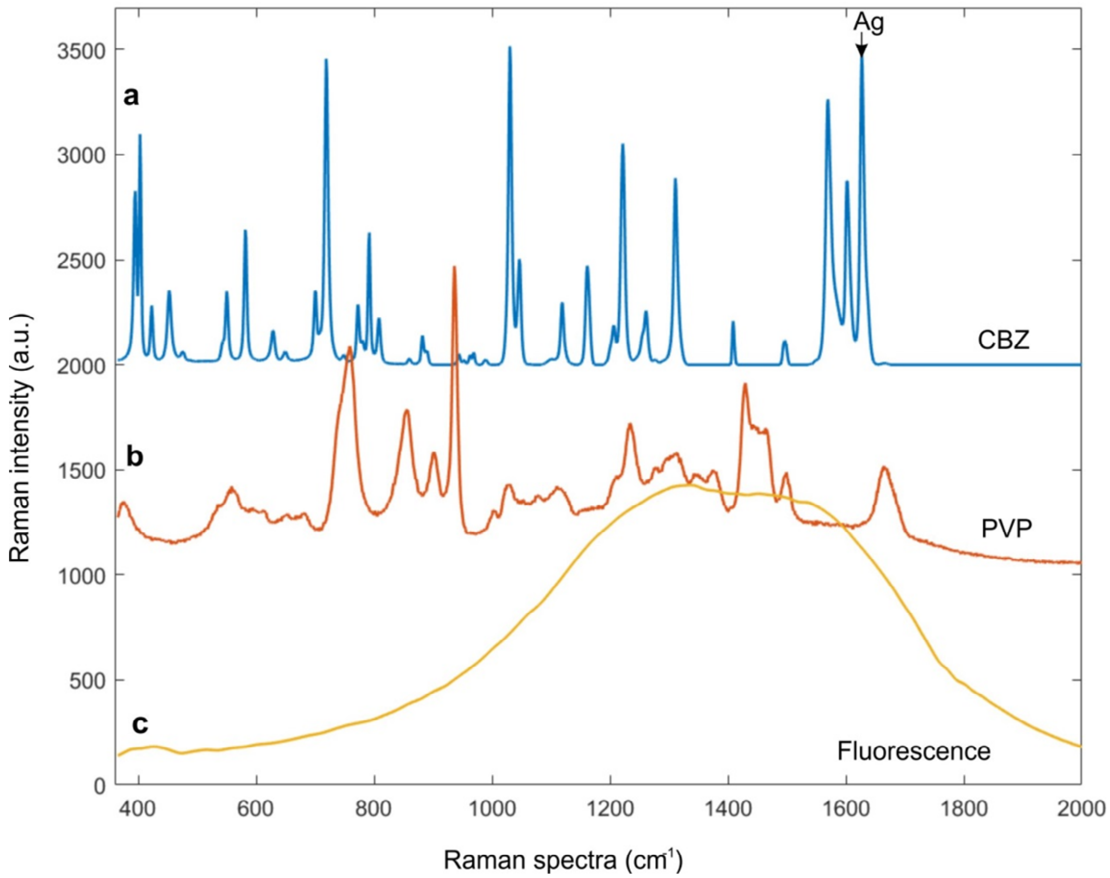

Supplementary Figure 22. Raman spectra of a pharmaceutical tablet. a, Raman spectrum of CBZD, **b,** Raman spectrum of PVP, **c,** fluorescence spectrum potentially due to magnesium stearate (tablet die) and impurities on the surface of the tablet.

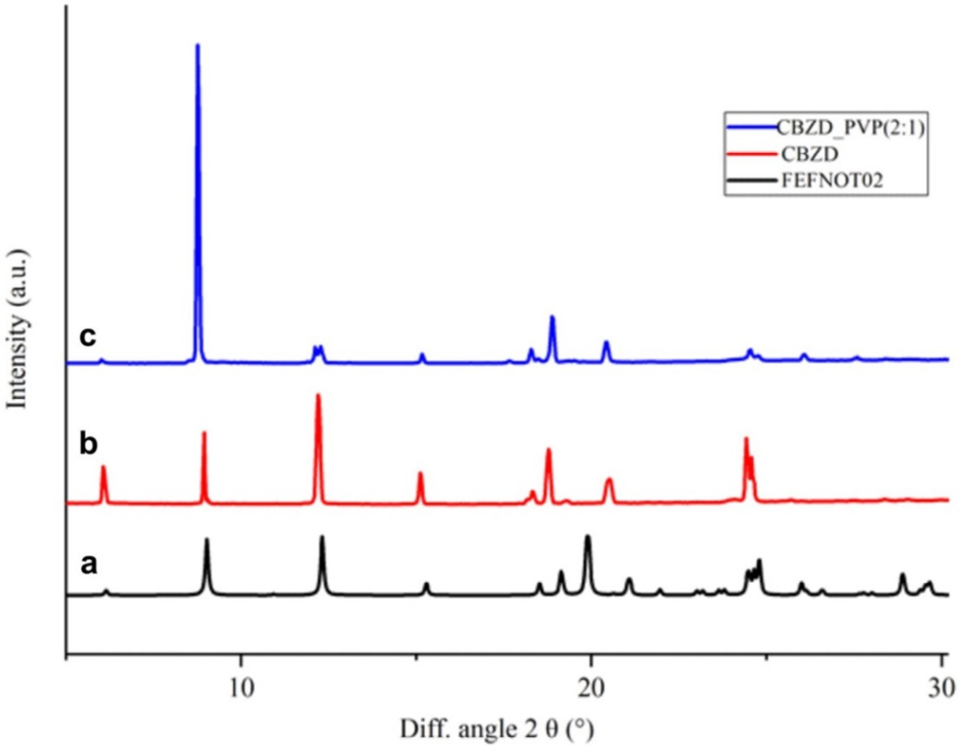

Supplementary Figure 23. XRPD diffractograms of CBZ. a, Calculated XRPD diffractogram of CBZD, **b,** experimentally obtained XRPD diffractorgam of CBZD and (**c)** CBZ and PVP (2:1).

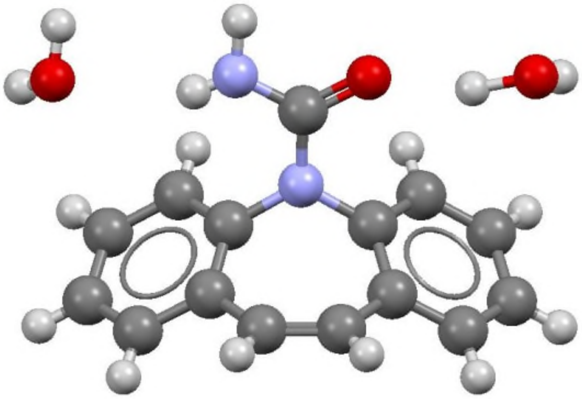

Supplementary Figure 24. Carbamazepine dihydrate molecular structure

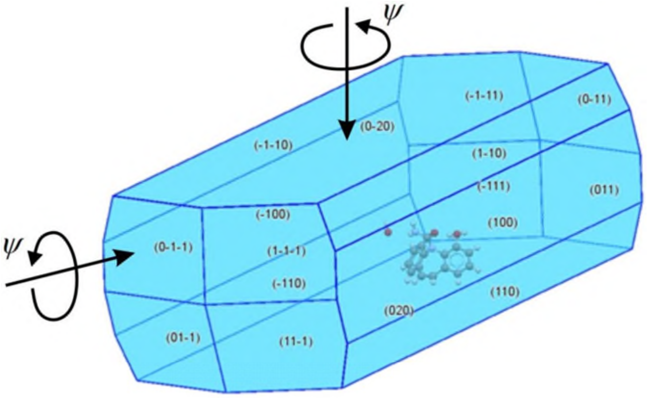

Supplementary Figure 25. The illustration of SAROM rotation experiments (see movie S4) applied to the CBZD. The predicted CBZD crystal morphology (CSD refcode FEFNOT02, monoclinic crystal system).

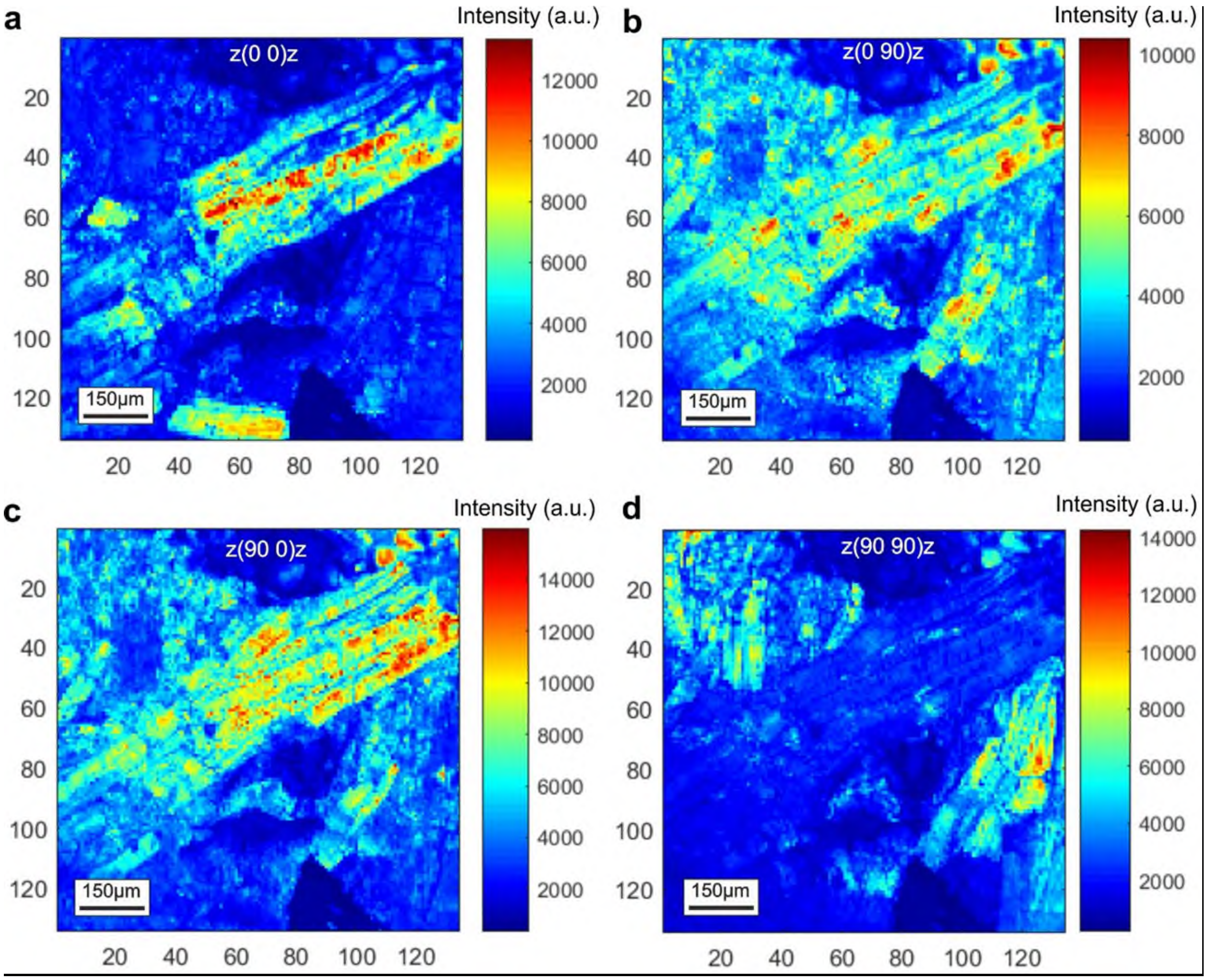

Supplementary Figure 26. Polarized Raman maps. Polarized Raman intensity maps of the surface of CBZD tablet obtained at polarization configurations $z(0\ 0)\bar{z}$ (**a**), $z(0\ 90)\bar{z}$ (**b**), $z(90\ 0)\bar{z}$ (**c**) and $z(90\ 90)\bar{z}$ (**d**).

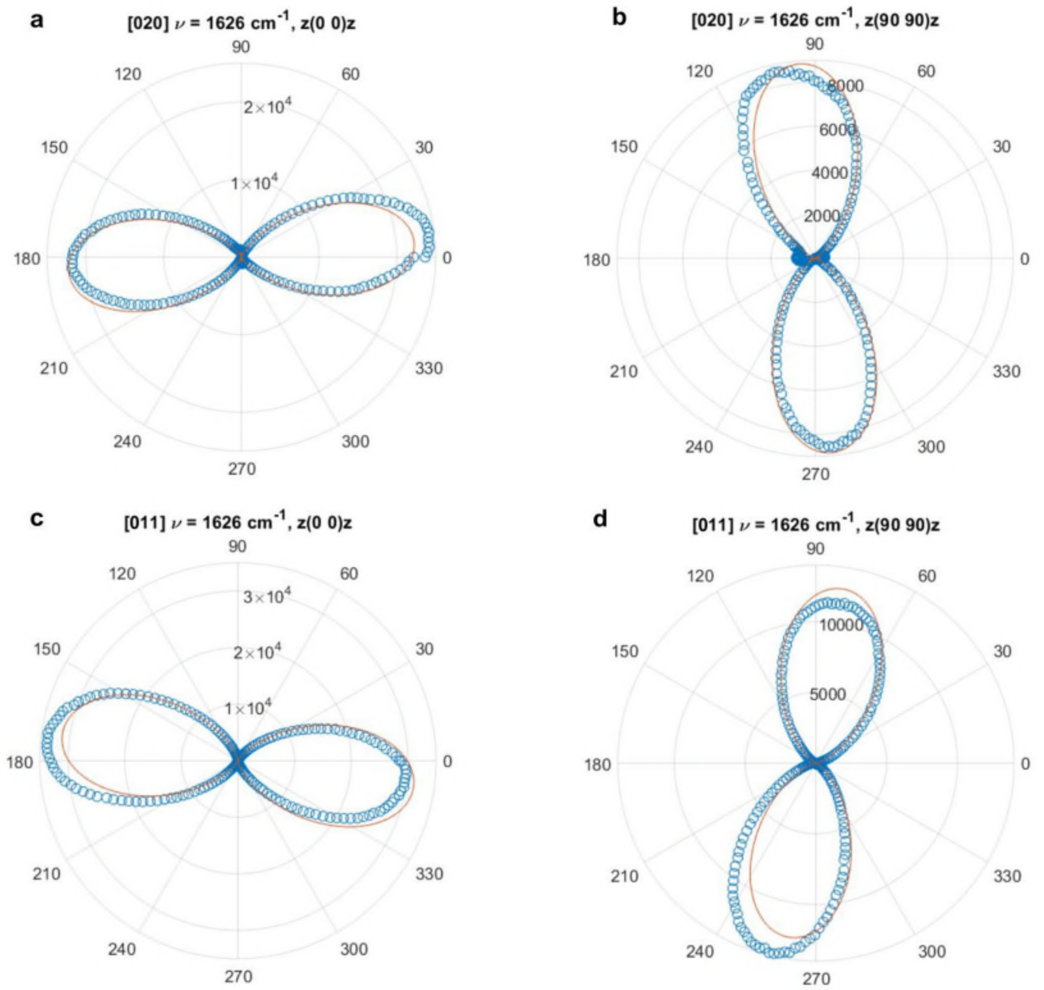

Supplementary Figure 27. Polarized Raman responses of CBZD. Comparison between theoretical (red line) and experimental (blue circles) responses of A_g mode of CBZD versus wafer rotation angle ψ plotted in polar coordinates for the monocrystal of CBZD around axis [020] (**a,b)** and [011] (**c,d)** at polarizer/analyzer orientations $z(0\ \ 0)\bar{z}$ (**a,c**), $z(90\ \ 90)\bar{z}$ (**b,d**).

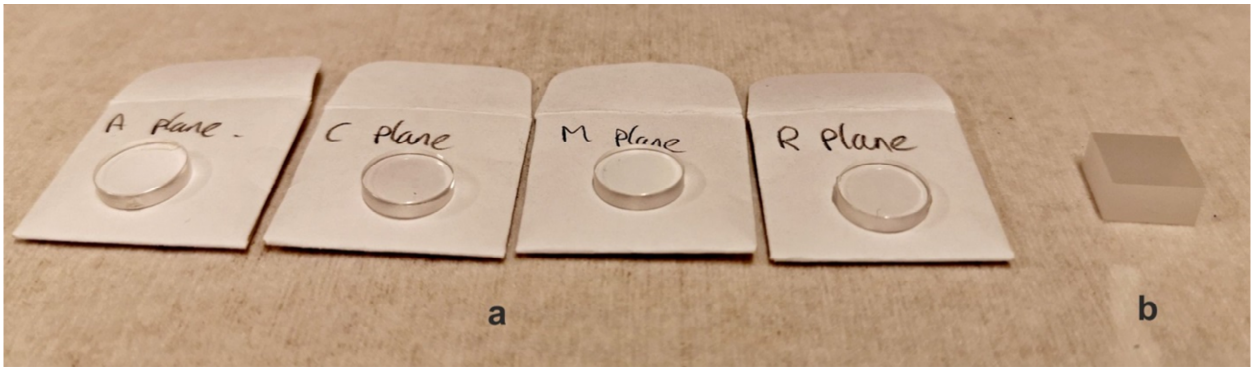

Supplementary Figure 28. Sapphire photographs. a, Photographs of the monocrystalline sapphire plates with cut at different surface planes, **b,** photograph of the polycrystalline sapphire sample.

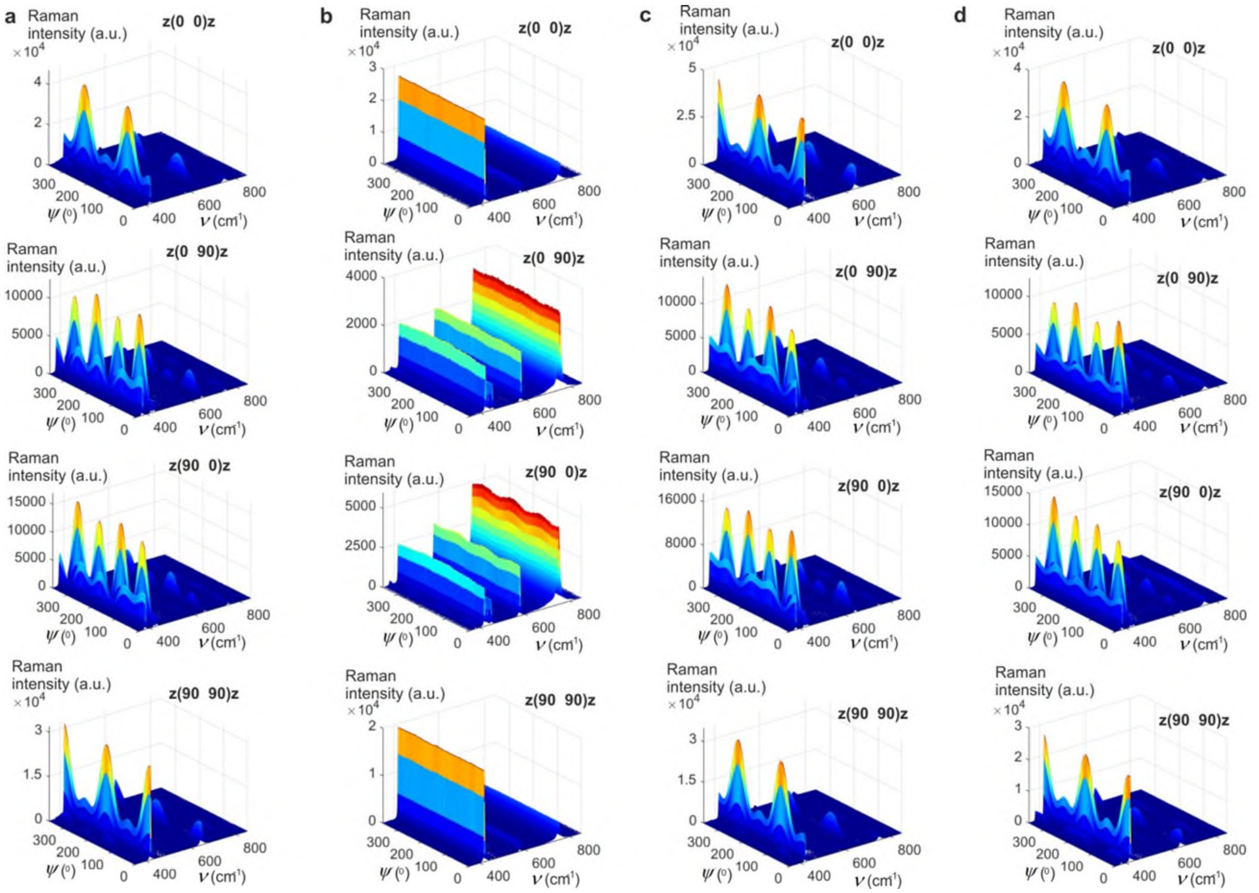

Supplementary Figure 29. Polarized Raman spectra of sapphire. 3D plots of the Raman spectra of sapphire for different on-axis polarization configurations versus sample rotation angle ψ for a- (**a**), c- (**b**), m- (**c**) and r- (**d**) planes.

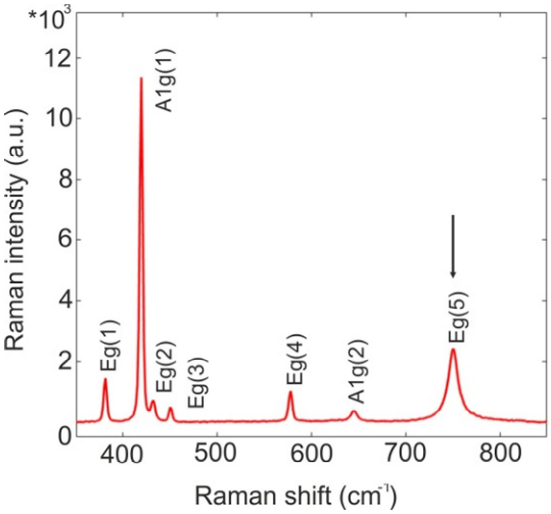

Supplementary Figure 30. Polarized Raman spectrum of sapphire with illustration of E_g and A_g modes.

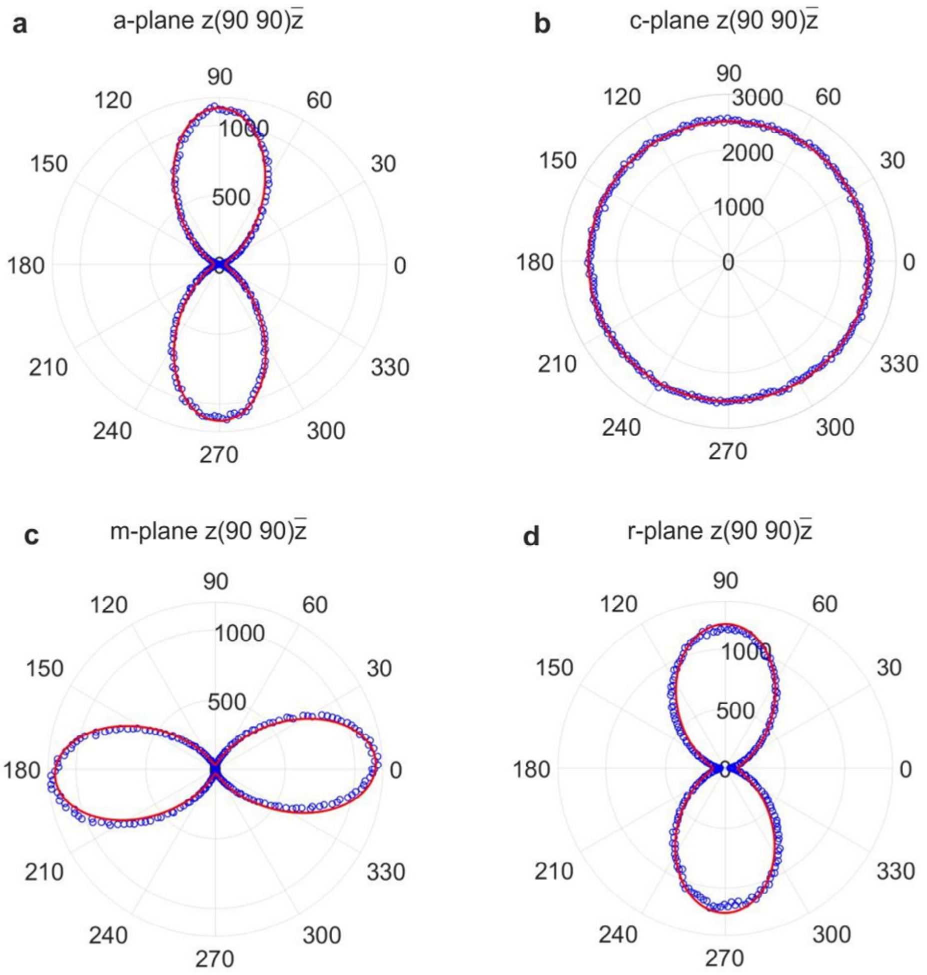

Supplementary Figure 31. Polarized Raman responses of sapphire. Comparison between theoretical (red line) and experimental (blue circles) responses of the $E_g(5)$ mode versus sapphire sample rotation angle ψ plotted in polar coordinates for polarization configuration $z(90\ \ 90)\bar{z}$ at a- (**a**), c- (**b**), m- (**c**) and r- (**d**) sapphire planes.

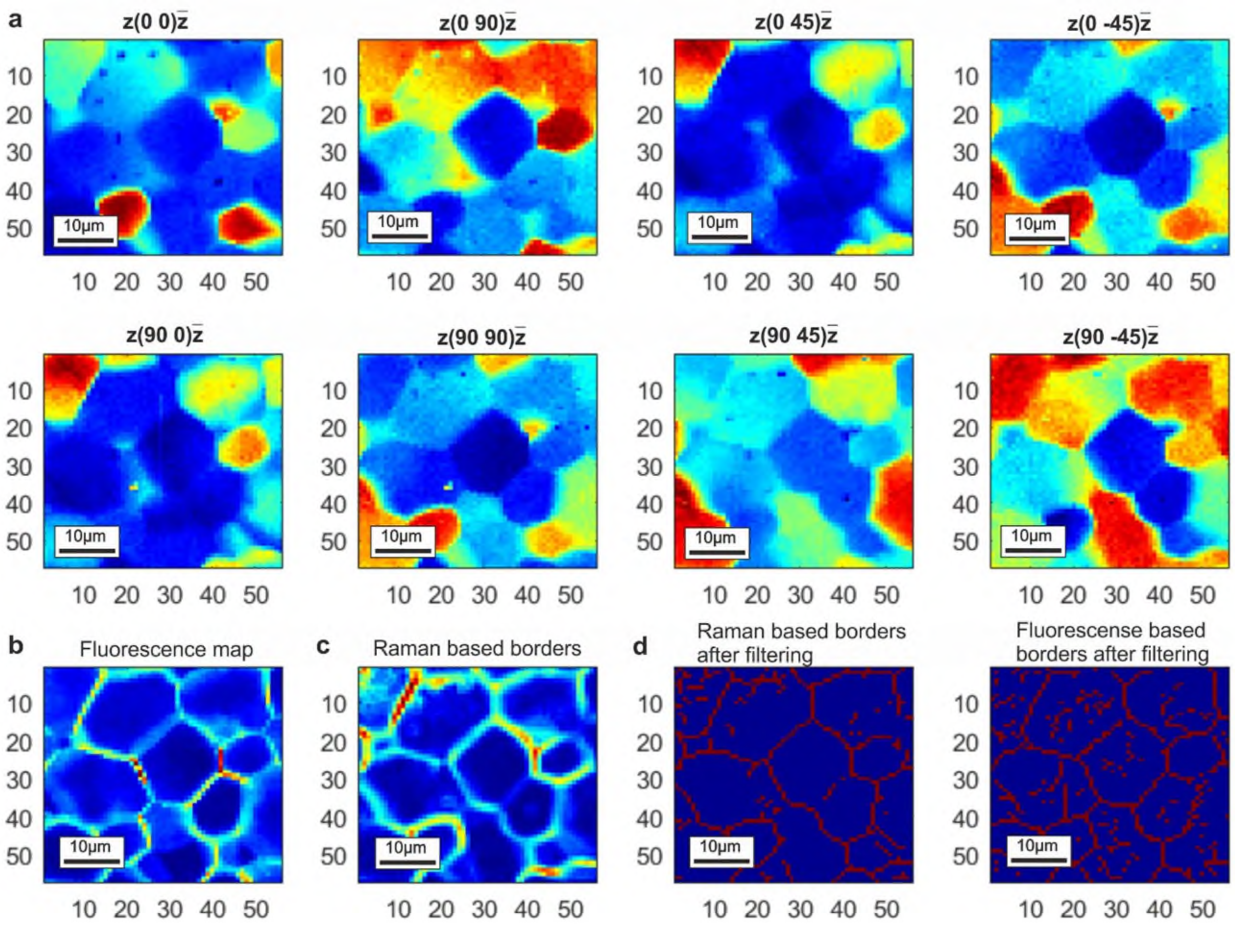

Supplementary Figure 32. SAROM mapping of polycrystalline sapphire. a, Polarized Raman maps of $E_g(5)$ mode at different polarization configurations, **b,** grained boundaries plotted based on fluorescence band, **c,** grained boundaries plotted based on Raman maps, **d,** comparison of segmentation results obtained on fluorescence and Raman responses.

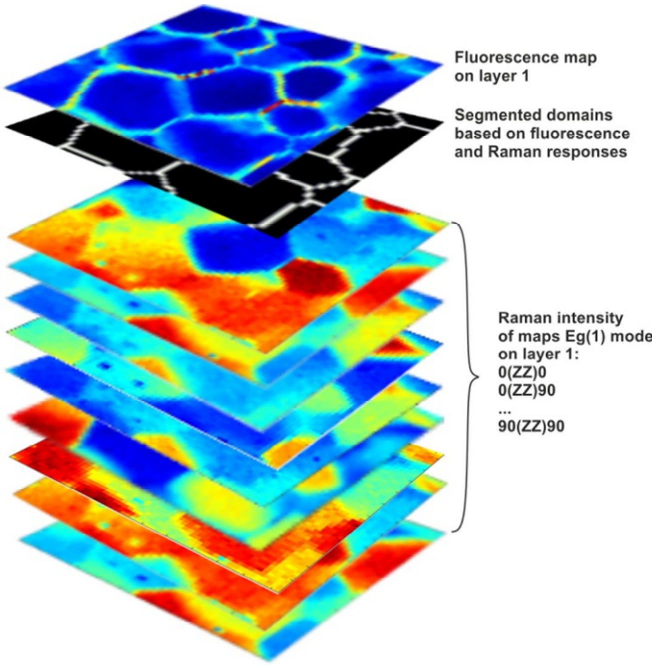

Supplementary Figure 33. Grain boundaries in polycrystalline sapphire. On top is grain boundary map based on the fluorescence, under it there is segmented domains based on combined fluorescence and Raman responses are shown, below are nine polarized Raman intensity maps for Eg(5) vibrational mode.

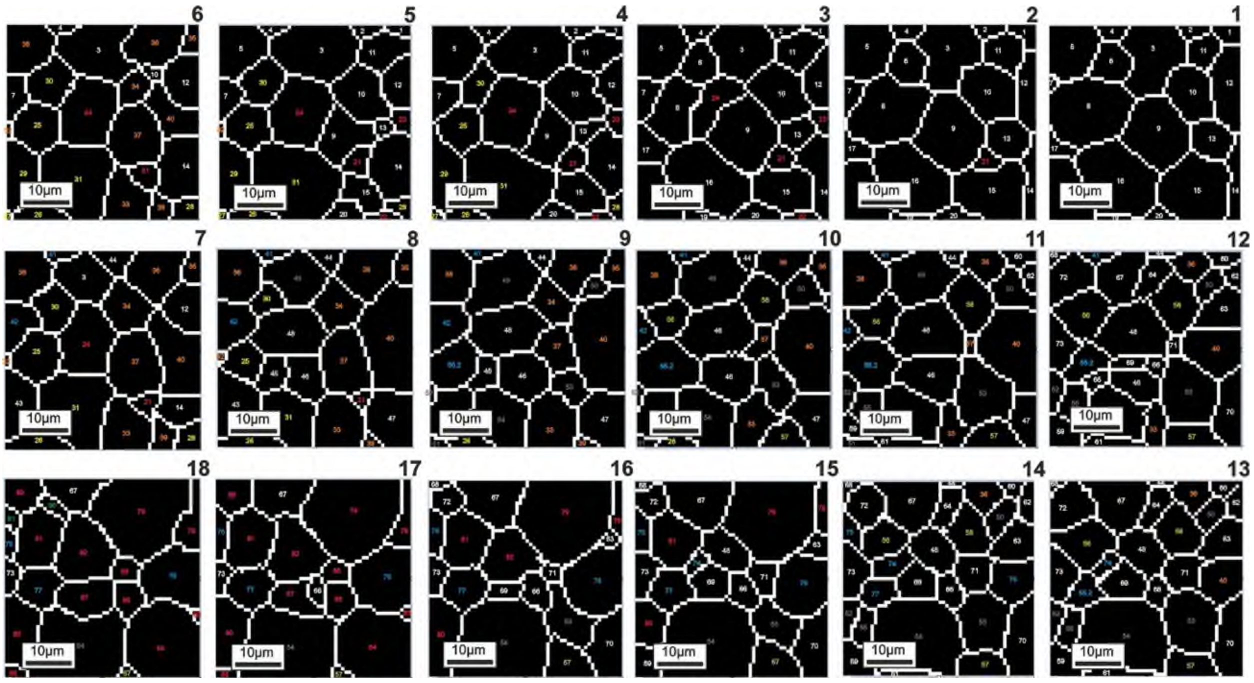

Supplementary Figure 34. Segmentation. Illustration of segmentation procedure applied to the nine acquired polarized Raman maps for 18 depth layers in 3D map, where segmented domains were plotted based on combined fluorescence and Raman responses.

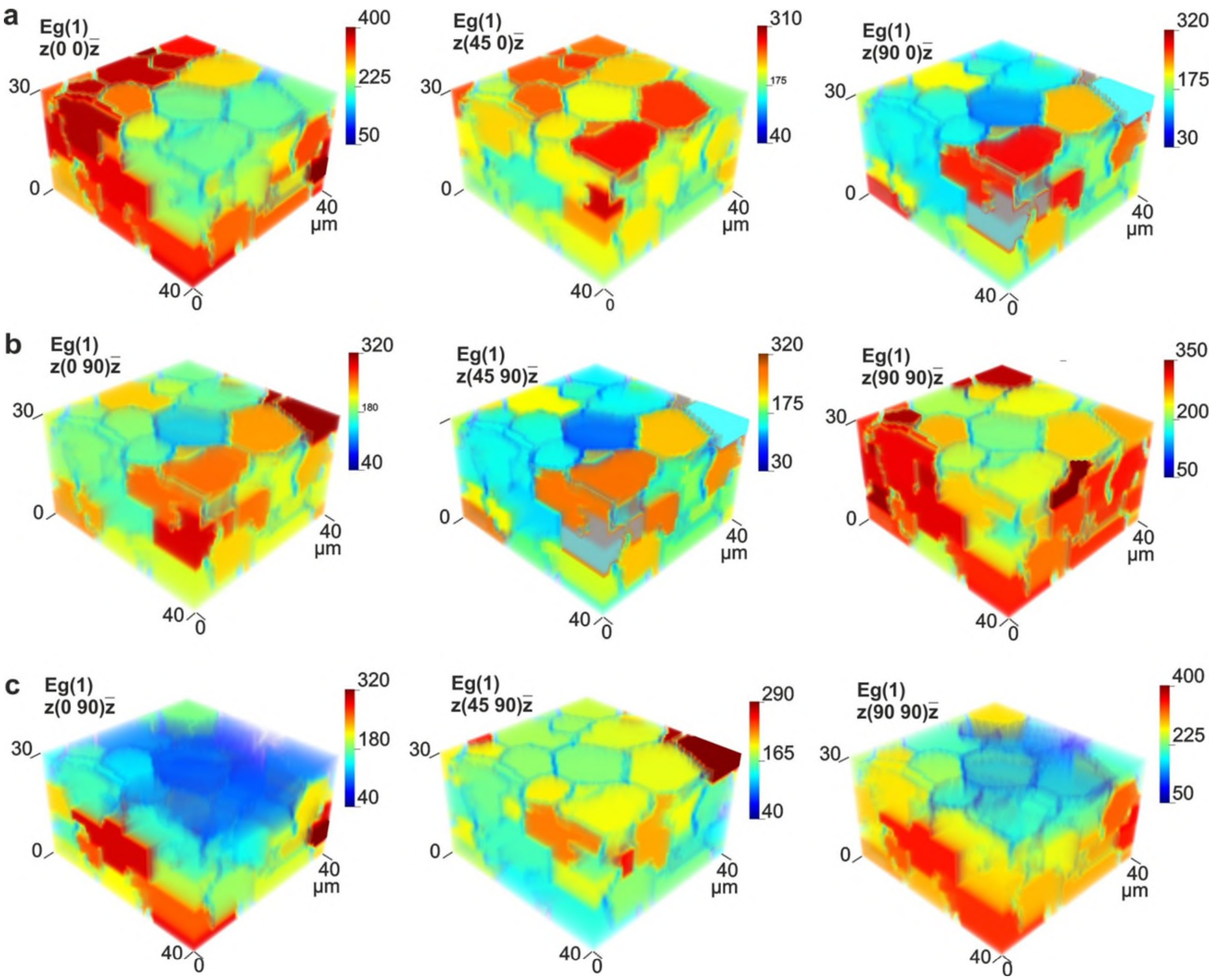

Supplementary Figure 35. Volumetric polarized Raman maps of polycrystalline sapphire for the nine polarization channels based on the Eg(5) mode. a, on-axis channels with analyzer orientation 0°, **b,** on-axis channels with analyzer orientation 90°, **c,** off-axis channels with analyzer orientation 90°. Intensity scale is in arbitrary units.

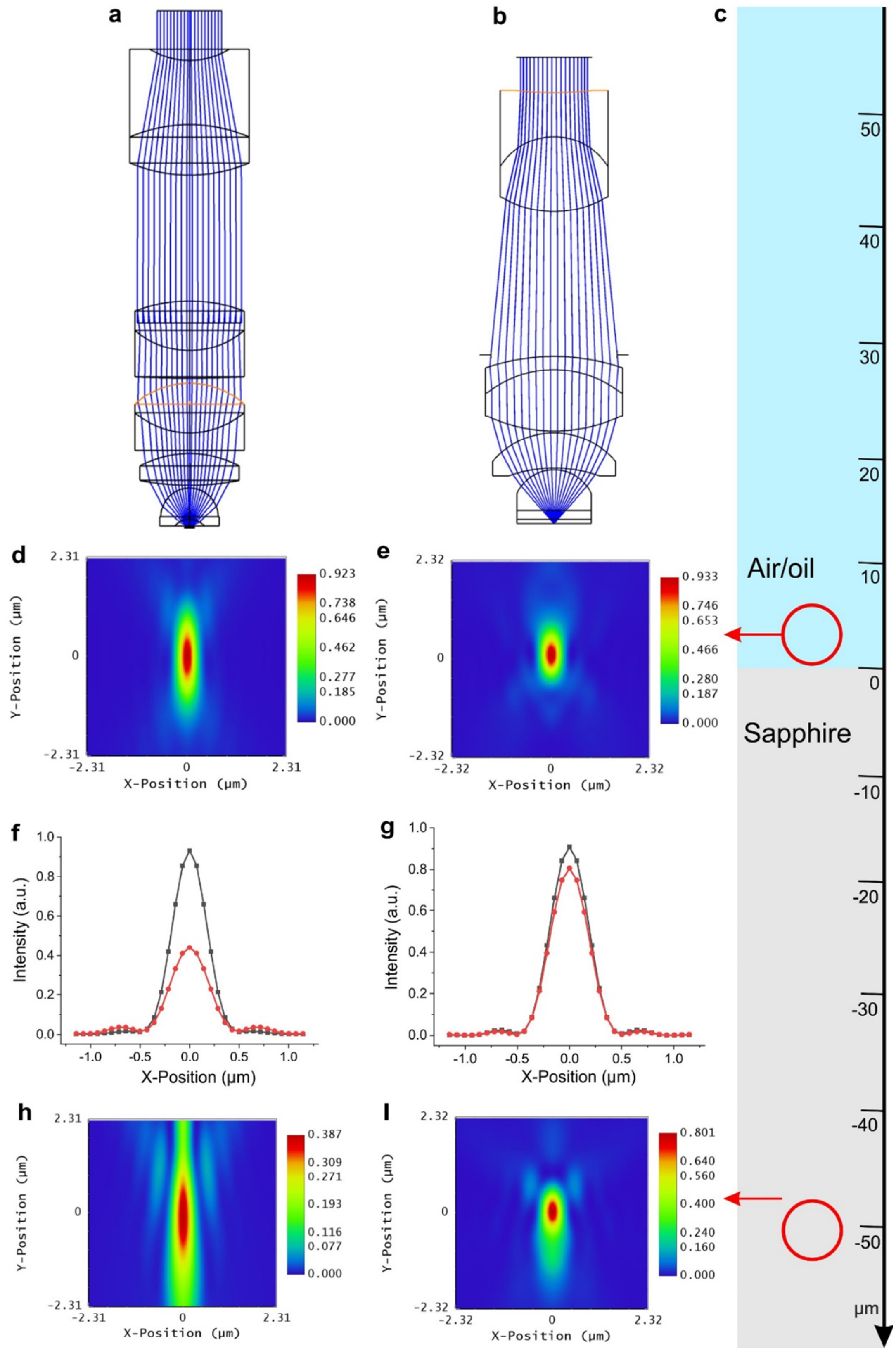

Supplementary Figure 36. Point spread function (PSF) of microscope objectives. a, an infinity-corrected, semi-apochromatic dry microscope objective (N.A.=0.85 at laser beam

diameter 6.5mm, magnification 50x, **b,** self-designed oil immersion objective for 3D measurements in Raman microscopy (NA=0.85 at laser beam diameter 8.8mm, magnification 45x), **c,** illustration of air-sapphire interface with regions of simulated PSF, **d,** axial PSF of dry objective in air, **e,** axial PSF of oil immersion objective in oil, **f,** cross sections of lateral PSF of dry objective in air (black line) and in the depth of sapphire at -50µm (red line), **g,** cross sections of lateral PSF of oil immersion objective in oil (black line) and in the depth of sapphire at -50µm (red line), **h,** axial PSF of dry objective in the depth of sapphire at -50µm, **i,** axial PSF of oil immersion objective in the depth of sapphire at -50µm.

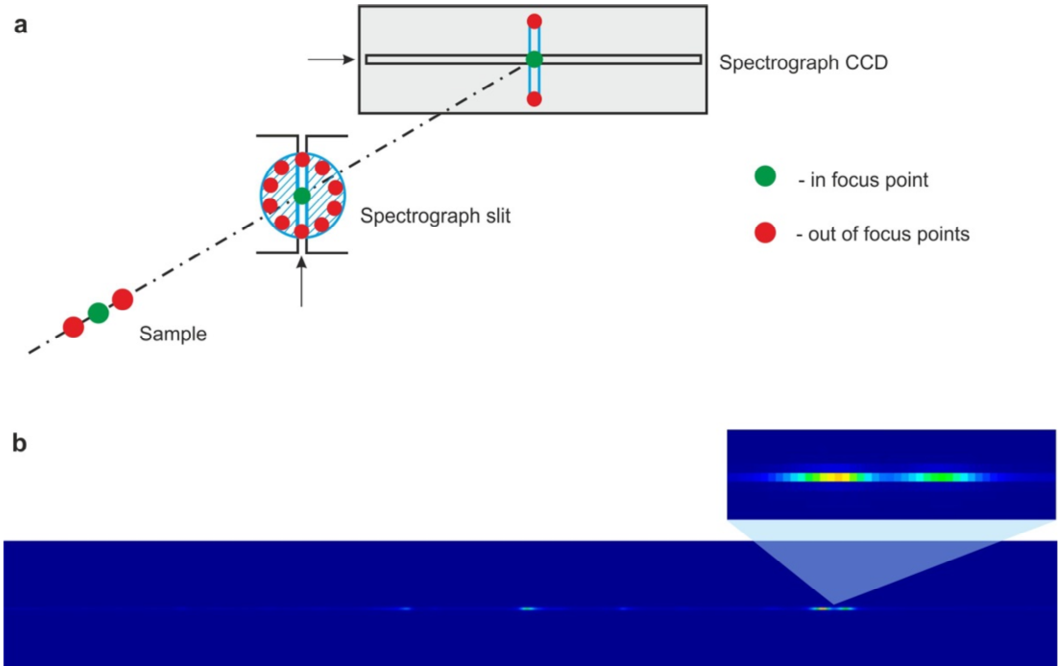

Supplementary Figure 37. Slit based confocal Raman setup. a, Slit based confocality principle used in aberration corrected Raman microscopy setup, **b,** the typical image from spectroscopic CCD Raman signal; zoomed region shows that spectrum compressed into one row on CCD sensor.

	Layers																	
Segments	1	2	3	4	5	6	7	8	9	10	11	12	13	14	15	16	17	18
1																		
2																		
3																		
4																		
5																		
6																		
7																		
8																		
9																		

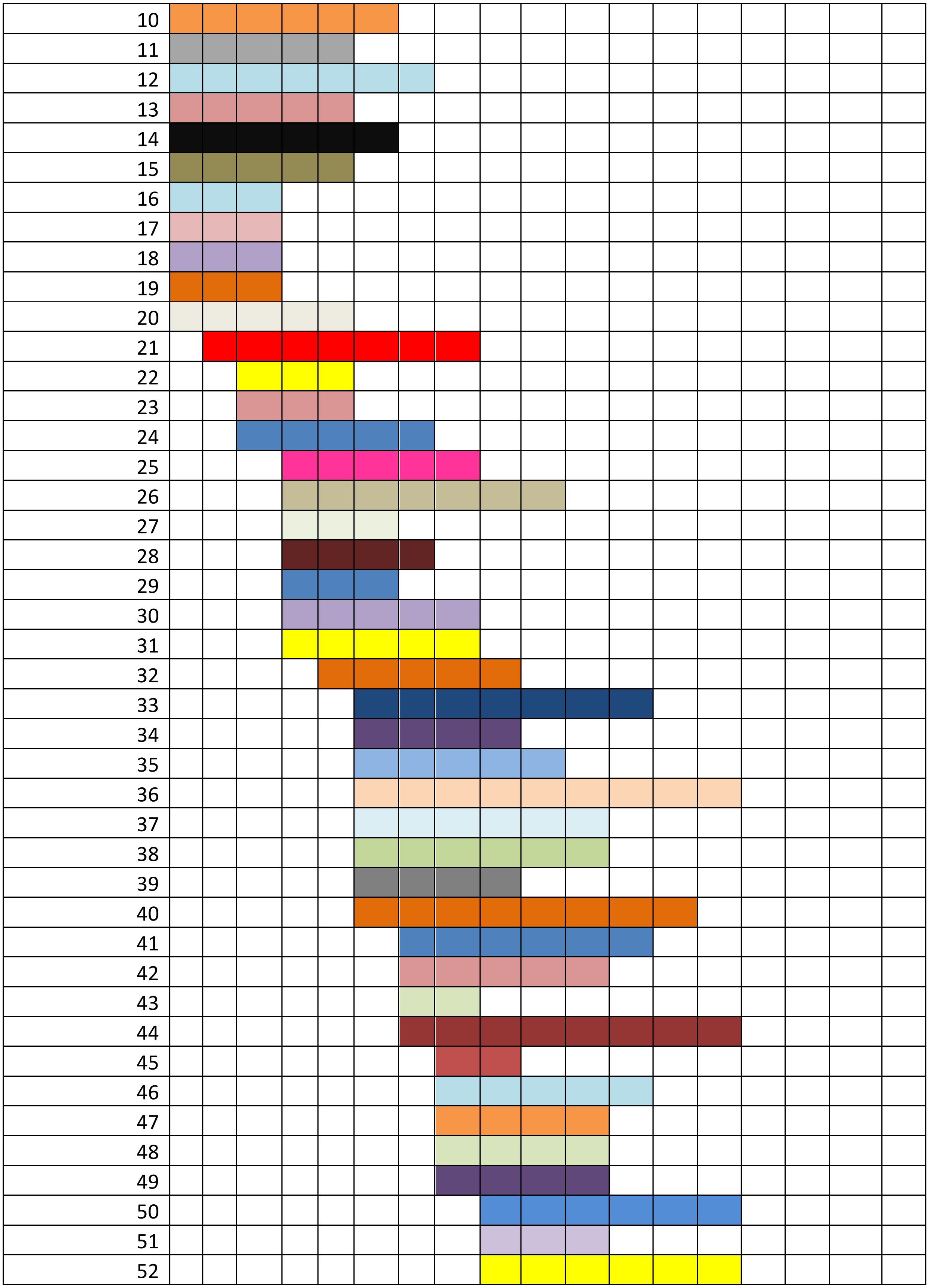
10
11
12
13
14
15
16
17
18
19
20
21
22
23
24
25
26
27
28
29
30
31
32
33
34
35
36
37
38
39
40
41
42
43
44
45
46
47
48
49
50
51
52

53																		
54																		
55																		
56																		
57																		
58																		
59																		
60																		
61																		
62																		
63																		
64																		
65																		
66																		
67																		
68																		
69																		
70																		
71																		
72																		
73																		
74																		
75																		
76																		
77																		
78																		
79																		
80																		
81																		
82																		
83																		
84																		
85																		
86																		
87																		
88																		
89																		
90																		
91																		

Supplementary Table 1. Sapphire segmentation results showing the propagation of domains from layer to layer in 3D map.

Supplementary References

1. Loudon, R. The Raman effect in crystals. *Adv. Phys.* **13**, 423–482 (1964).
2. Pezzotti, G. & Zhu, W. Resolving stress tensor components in space from polarized Raman spectra: Polycrystalline alumina. *Phys. Chem. Chem. Phys.* **17**, 2608–2627 (2015).
3. Kranert, C., Sturm, C., Schmidt-Grund, R. & Grundmann, M. Raman Tensor Formalism for Optically Anisotropic Crystals. *Phys. Rev. Lett.* **116**, 1–5 (2016).
4. Kranert, C., Sturm, C., Schmidt-Grund, R. & Grundmann, M. Raman tensor elements of β-Ga2O3. *Sci. Rep.* **6**, 1–9 (2016).
5. Is, W. & Analysis, P. Raman Polarization Measurements : Keeping Track of the Instrumental Components ' Behavior Raman Polarization Measurements : Keeping Track of the Instrumental Components ' Behavior Raman Polarization Measurements : Keeping Track of the Instrumental Compone. 1–9 (2018).
6. Mizoguchi, K. & Nakashima, S. I. Determination of crystallographic orientations in silicon films by Raman-microprobe polarization measurements. *J. Appl. Phys.* **65**, 2583–2590 (1989).
7. Becker, M., Scheel, H., Christiansen, S. & Strunk, H. P. Grain orientation, texture, and internal stress optically evaluated by micro-Raman spectroscopy. *J. Appl. Phys.* **101**, (2007).
8. Hielscher, R. & Schaeben, H. A novel pole figure inversion method: specification of the MTEX algorithm. *J. Appl. Crystallogr.* **41**, 1024–1037 (2008).
9. Mainprice, D., Bachmann, F., Hielscher, R. & Schaeben, H. Descriptive tools for the analysis of texture projects with large datasets using MTEX : strength, symmetry and components. *Geol. Soc. London, Spec. Publ.* **409**, 251–271 (2015).
10. Harris, R. K. *et al.* Structural Studies of the Polymorphs of Carbamazepine, Its Dihydrate, and Two Solvates. *Org. Process Res. Dev.* **9**, 902–910 (2005).
11. Strachan, C. J., Howell, S. L., Rades, T. & Gordon, K. C. A theoretical and spectroscopic study of carbamazepine polymorphs. *J. Raman Spectrosc.* **35**, 401–408 (2004).
12. Munisso, M. C., Zhu, W. & Pezzotti, G. Raman tensor analysis of sapphire single crystal and its application to define crystallographic orientation in polycrystalline alumina. *Phys. status solidi* **246**, 1893–1900 (2009).
