# Supplementary material for: Fast and quantitative 2D and 3D orientation mapping using Raman microscopy
Source: Nat Commun. 2019 Dec 5;10:5555. doi: 10.1038/s41467-019-13504-8 (PMC6895231; doi:10.1038/s41467-019-13504-8)
Supplement: Supplementary file 3 — Description of additional supplementary files [file 41467_2019_13504_MOESM3_ESM.docx]

**Description of Additional Supplementary Files**

File Name: Supplementary Movie 1.

Description: Demonstration of polycrystalline Si mapping with simultaneous registration of

several polarized channels in SAROM software.

File Name: Supplementary Movie 2.

Description: Experimental demonstration of intensity variation in polarized Raman spectral

responses versus monocrystalline CBZD drug particle rotation.

File Name: Supplementary Movie 3.

Description: Experimental demonstration of intensity variation in polarized Raman spectral responses versus monocrystalline sapphire plates rotation.

File Name: Supplementary Movie 4.

Description: 3D-SAROM orientation map of polycrystalline sapphire.

File Name: Supplementary Data 1.

Description: Polarized Raman maps of polycrystalline Si obtained at multiple polarization

configurations in SAROM (data used in Supplementary Figure 16).

File Name: Supplementary Data 2.

Description: Orientation map of polycrystalline Si obtained by SAROM (data used in Fig. 3).

File Name: Supplementary Data 3.

Description: Orientation map of polycrystalline Si obtained by EBSD (data used in Fig. 3).

File Name: Supplementary Data 4.

Description: Orientation map of CBZD tablet formulation obtained by SAROM (data used in Fig.4).

File Name: Supplementary Data 5.

Description: Orientation map of polycrystalline sapphire obtained by SAROM (data used in Fig. 5).
